# Computational Lipidology: Predicting Lipoprotein Density Profiles in Human Blood Plasma

**DOI:** 10.1371/journal.pcbi.1000079

**Published:** 2008-05-23

**Authors:** Katrin Hübner, Thomas Schwager, Karl Winkler, Jens-Georg Reich, Hermann-Georg Holzhütter

**Affiliations:** 1 Computational Systems Biochemistry, Institute of Biochemistry, Charité-Universitätsmedizin Berlin, Germany; 2 Bioinformatics Group, Max Delbrück Center for Molecular Medicine Berlin-Buch, Germany; 3 Department of Clinical Chemistry, University Hospital Freiburg, Germany; University of California San Diego, United States of America

## Abstract

Monitoring cholesterol levels is strongly recommended to identify patients at risk for myocardial infarction. However, clinical markers beyond “bad” and “good” cholesterol are needed to precisely predict individual lipid disorders. Our work contributes to this aim by bringing together experiment and theory. We developed a novel computer-based model of the human plasma lipoprotein metabolism in order to simulate the blood lipid levels in high resolution. Instead of focusing on a few conventionally used predefined lipoprotein density classes (LDL, HDL), we consider the entire protein and lipid composition spectrum of individual lipoprotein complexes. Subsequently, their distribution over density (which equals the lipoprotein profile) is calculated. As our main results, we (i) successfully reproduced clinically measured lipoprotein profiles of healthy subjects; (ii) assigned lipoproteins to narrow density classes, named *high-resolution density sub-fractions* (hrDS), revealing heterogeneous lipoprotein distributions within the major lipoprotein classes; and (iii) present model-based predictions of changes in the lipoprotein distribution elicited by disorders in underlying molecular processes. In its present state, the model offers a platform for many future applications aimed at understanding the reasons for inter-individual variability, identifying new sub-fractions of potential clinical relevance and a patient-oriented diagnosis of the potential molecular causes for individual dyslipidemia.

## Author Summary

Lipids such as cholesterol and triglycerides, which are synthesized in the body or taken up by food, are indispensable for each cell of the human body. They are transported in blood plasma among the various tissues by so-called lipoproteins, which differ in size as well as in their composition of lipids and proteins. Changes in the amount of certain lipoprotein fractions are considered a major risk factor for atherosclerosis and cardiovascular diseases (CVD)—the main cause of death in the western states. To identify patients at risk for CVD, major lipoprotein classes (“bad” LDL, “good” HDL) are routinely monitored in clinical practice (which equals the lipoprotein profile). In this paper, we present a mathematical model that allows us to calculate lipoprotein profiles by computer and to simulate how the numerous biochemical processes involved in the metabolism of plasma lipoproteins influence the lipoprotein profile. Our simulations successfully reproduce clinically measured lipoprotein profiles of healthy subjects and patients with a defined lipid disorder (dyslipidemia). Calculation of a lipoprotein profile that fits best to the profile measured in individual patients opens the possibility of diagnosing potential molecular causes for dyslipidemia. The results of our model calculations also suggest the existence of lipoprotein sub-fractions which hitherto remained unrecognized in routinely performed separation of lipoprotein fractions. If this finding could be validated in forthcoming experimental investigations, it might help to define better patient-specific risk predictors of CVD.

## Introduction

Lipids are almost insoluble in aqueous media such as blood plasma and thus transported among the various tissues by water-soluble complexes called lipoproteins (LP). Elucidating the kinetic mechanisms involved in the formation, degradation and mutual interconversion of plasma lipoproteins is of high medical relevance as long-term perturbations of the lipoprotein distribution are considered the primary risk factor for atherosclerosis and cardiovascular diseases—the main cause of death in the western states [1]. Each lipoprotein complex contains a discrete number of apolipoproteins (e.g. apolipoprotein B-100) and lipid molecules (e.g. triglycerides, cholesterol), the number of which may vary between one and several hundreds or thousands, respectively. Basically, an enormous lipoprotein heterogeneity results from all possible stoichiometric combinations of lipid and apolipoprotein molecules. Despite this fact, for almost half a century, lipoproteins have usually been grouped into density classes named chylomicrons, VLDL, IDL, LDL, and HDL (very low-, intermediate-, low-, and high-density lipoproteins, respectively), separated by, for example, ultracentrifugation from blood plasma [2]. Accordingly, mathematical models of the systemic lipoprotein metabolism hitherto have considered lipoprotein density classes (compartments) to be dynamic variables of the system. The phenomenological transition rates between these compartments are usually determined by radioactive or stable isotope tracer experiments (for reviews see [3]–[5]). Except for the comprehensive compartment model proposed by Knoblauch et al. [6], compartment models have focused on specific parts of the lipoprotein metabolism based on kinetic measurements with, for example, labeled apoA-I [7]–[10], apoA-II [11], or apoB-100 [12]–[14]. Compartment models may provide a useful phenomenological description of the lipoprotein dynamics; however, they have some serious limitations. First, they neglect the possible heterogeneity of lipoproteins. Actually, a single density class comprises a huge number of lipoprotein complexes differing in their amount of lipids and proteins—an important fact that could be of relevance for the medical interpretation of lipoprotein density profiles. Second, the transition of a lipoprotein from one density class into another is not a single process but is accomplished in a series of successive elementary reactions in which, for example, triglycerides are removed, cholesterols are taken up from tissues, and apolipoproteins are exchanged. Therefore, phenomenological inter-compartment transition rates can hardly be related to the rate of the underlying molecular processes. To overcome these limitations of compartment models, we propose here a novel approach. It consists of the establishment of kinetic equations governing the temporal changes of individual lipoprotein complexes. Hence, in our modeling approach, the number of dynamic variables is in principle given by the number of different lipoprotein complexes that can be formed from a given number of apolipoproteins and lipids. From the calculated set of individual lipoprotein complexes in the system we can compute the distribution of lipoproteins over an arbitrary number of predefined density classes (the lipoprotein profile). In particular, we can compute such profiles over commonly defined density classes and compare them with experimental profiles obtained from normolipidemic subjects. Choosing a larger set of narrow density classes and subdividing the calculated lipoprotein distribution in, as we call it, *high-resolution density sub-fractions* (hrDS), we observe a remarkable heterogeneity of the lipoprotein distribution within commonly defined density classes. Finally, we calculate lipoprotein profiles associated with a disorder in one of the underlying molecular processes of the lipoprotein metabolism. In these simulations of pathological situations we altered the rate constants of the LDL receptor-mediated lipoprotein uptake, lipoprotein lipase, and ABCA1-mediated cholesterol transport.

## Results

### The Model

We avoid usage of predefined density classes but characterize lipoproteins regarding their protein and lipid composition. As described below, the model takes into account essential lipoprotein constituents and processes involved in the lipoprotein metabolism in human blood plasma. However, for the sake of numerical tractability we reduced the number of lipoprotein components to a manageable set and simplified the kinetic processes.

#### Lipoprotein components

The lipoprotein complexes considered in the model are composed of three different types of apolipoproteins and lipids abbreviated as A, B, F, and C, T, P, respectively. The protein components A and B are thought to represent apoA-I (P02647, ENSG00000215756) as the primary protein constituent of HDL and apoB-100 (P04114, ENSG00000084674) as the characteristic apolipoprotein of VLDL, IDL, and LDL, respectively. We ignore apoB-48 and the related lipoprotein complexes (chylomicrons), which are rapidly formed and degraded within several hours after food intake. Each lipoprotein is either equipped with component A or component B. Thus, we will use the terms *A-particles* and *B-particles* in the following, respectively. All other apolipoproteins are lumped together into the protein component F. The lipid components C, T, and P represent total cholesterol (free cholesterol and cholesteryl esters), triglycerides, and phospholipids, respectively. The dynamics of phospholipids (P) is not explicitly modeled. Instead, the number of phospholipid molecules in an individual lipoprotein complex is calculated such that, together with the apolipoproteins, full occupancy of the lipoprotein surface is achieved (see Dataset S1 for details of calculation).

The component's densities vary between 1.35 and 0.886 g/ml for apolipoproteins and triglycerides, respectively. The possible number of lipid molecules may go up to several thousands. This results in a considerably diversity of lipoprotein complexes in the system. With the maximal number of molecules for each component given in Table 1, we would get 8.0×10^8^ lipoprotein complexes in total. Thus, to keep the model tractable we refrained from considering the actual number of molecules for total cholesterol (C) and triglycerides (T). Instead, their content was quantified in terms of lipid packages. The package size has to be chosen carefully to avoid sparsely occupied density ranges. In the calculations below, lipid packages of C and T comprise 2 molecules in A-particles and 20 molecules in B-particles, respectively.

**Table 1:** Composition properties of lipoprotein complexes.

Lipoprotein species		Component Particle Number				
		A	B	F	C	T
A particle	Min	1	0	0	0	0
	Max	4	0	15	300	50
	Initial	1	0	0	10	0
B particle	Min	0	1	0	0	0
	Max	0	1	15	5000	10,000
	Initial	0	1	10	2000	10,000

Min and Max represent the lower and upper limit of component's number, respectively. Initial displays the initial composition of newly synthesized lipoprotein particles. Lipid (C,T) package sizes were defined as 2 and 20 molecules in A- and B-particles, respectively. Thus, in terms of packages, initial A-particles contain 5 packages of cholesterol molecules, and initial B-particles contain 100 cholesterol and 500 triglyceride packages.

#### Kinetic processes

**Figure 1:**
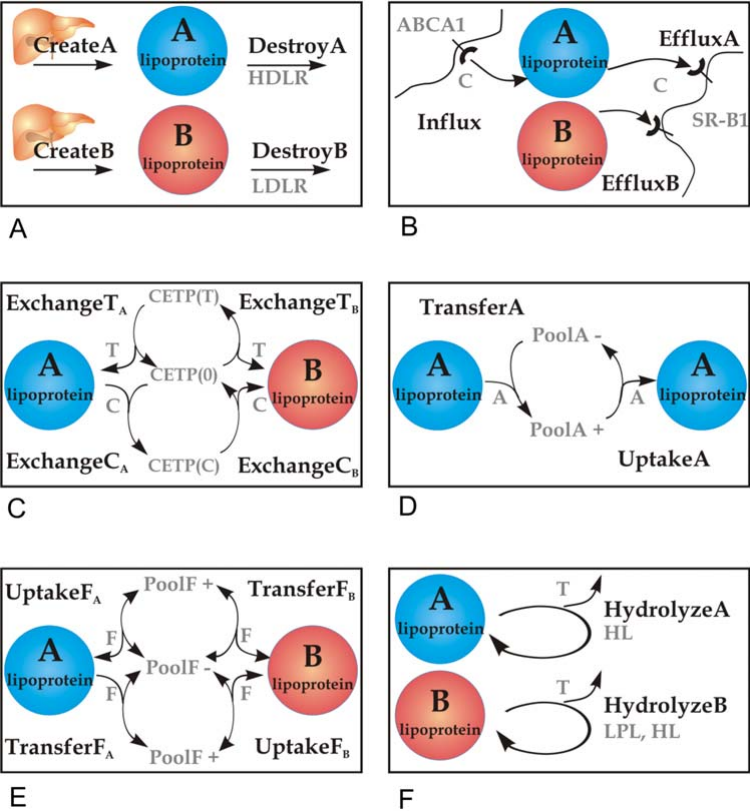
Schematic representation of the kinetic processes modeled. (A) Synthesis of A- and B-particles and degradation via HDL and LDL receptors, respectively. (B) Influx of peripheral cholesterol (“C”) into A-particles via the ATP-binding cassette A1 (ABCA1) receptor and selective efflux of cholesteryl ester (“C”) by the scavenger receptor B1 (SR-B1). (C) Elementary processes of the cholesteryl ester transfer protein (CETP) mediating the exchange of triglycerides (“T”) and cholesteryl ester (“C”) between lipoprotein components. CETP(0), CETP(T), and CETP(C) represent non-lipid–bound, T- and C-loaded forms of CETP, respectively. (D, E) Exchange of apolipoproteins (“A,” “F”) among lipoprotein complexes via plasma pools (PoolA, PoolF). (F) Hydrolysis of triglycerides (“T”) from A- and B-particles by hepatic and/or lipoprotein lipase (HL, LPL).

From the reactions reported to affect the lipoprotein metabolism in human blood plasma we selected 20 elementary processes. As we lump together free cholesterol and cholesteryl ester into one component (total cholesterol), esterification of free cholesterol by the lecithin-cholesterol acyltransferase (LCAT, EC 2.3.1.43, P04180, ENSG00000213398) is not considered. A summary of the kinetic processes included in the model and their physiological meaning is given in Dataset S2. For a schematic representation see Figure 1. We grouped the processes into six categories: (1) Birth and death: the total amount of lipoprotein complexes is the result of de novo synthesis by the liver and the receptor-mediated uptake of whole particles from the blood by tissue cells (Figure 1A). Separate kinetic parameters are used for the generation and elimination of A- and B-particles. The initial composition of newly synthesized particles was set to fixed values given in Table 1. (2) Lipoprotein-tissue exchange: besides the synthesis and uptake of whole lipoprotein complexes (see Category 1), individual components are selectively altered by exchange processes with various tissue cells. The uptake of peripheral cholesterol by A-particles and the release of cholesteryl esters from both particle species are taken into account (Figure 1B). (3) Inter-lipoprotein exchange of neutral lipids among lipoproteins is mediated by the cholesteryl ester transfer protein (CETP; P11597, ENSG00000087237), which transfers preferentially cholesteryl esters from A- to B-particles and triglycerides and vice versa. To model this transfer, we introduce a non-lipid bound form of this carrier protein (called CETP(0) in the model) that can be loaded either with C (called CETP(C)) or T (called CETP(T)) which shuttles between A- and B-particles (Figure 1C). The transfer of triglycerides between B-particles is included as well and defined as a separate process. (4) Exchange of apolipoprotein A. The transfer of apolipoproteins that can be exchanged among lipoprotein complexes is modeled by decomposing it into (i) a release step from a lipoprotein complex into a common plasma pool of free apolipoprotein and (ii) an uptake process from this pool into a lipoprotein complex. The transfer process for the protein component A is restricted to A-particles and is thought to describe the re-modeling of apoA-containing HDL (Figure 1D). (5) Exchange of apolipoproteins F. The transfer of those apolipoproteins, mostly apoE (P02649, ENSG00000130203) and apoC, lumped together into the component F may take place between arbitrary lipoprotein complexes (Figure 1E). (6) Enzymatic conversion. One central enzymatic process effecting the re-modeling of lipoproteins is the hydrolysis of lipoproteins' triglycerides and phospholipids. This process is catalyzed by the lipoprotein lipase (LPL; EC 3.1.1.34, P06858, ENSG00000175445) or the hepatic lipase (HL; EC 3.1.1.3, P11150, ENSG00000166035), which are treated in the model as two separate processes (Figure 1F).

### Stochastic Versus Deterministic Model Simulations

We modeled and simulated the system of lipoproteins by two different approaches, a stochastic and a deterministic one. For a detailed description the reader is referred to the Materials and Methods section. In brief, in the stochastic simulation the calculation of stationary lipoprotein distributions was performed by simulating the Master equation by means of the Gillespie algorithm until a stationary state was reached. The deterministic model (Equation 5) governing the lipoprotein concentrations comprises as many kinetic equations as there are different lipoprotein complexes. However, the concentrations of most complexes are practically zero because the elementary processes are highly specific. Moreover, the numerical solution of very large systems of equations poses serious numerical problems.

For the stochastic simulation of the Master equation this enormous complexity is not a problem as the stochastic trajectories generated by the Gillespie algorithm are located in a very small region within the space of all possible lipoprotein complexes. Hence, Gillespie's direct method does not suffer from the type of combinatorial explosion as the deterministic approach. Furthermore, using the stochastic simulation algorithm instead of the system of differential equations permits to deal with small package sizes for the lipid components, i.e. the number of lipid molecules per package. Small package sizes are needed to achieve a sufficiently high coverage of physiologically relevant density intervals containing a number of different lipoprotein complexes.

**Figure 2:**
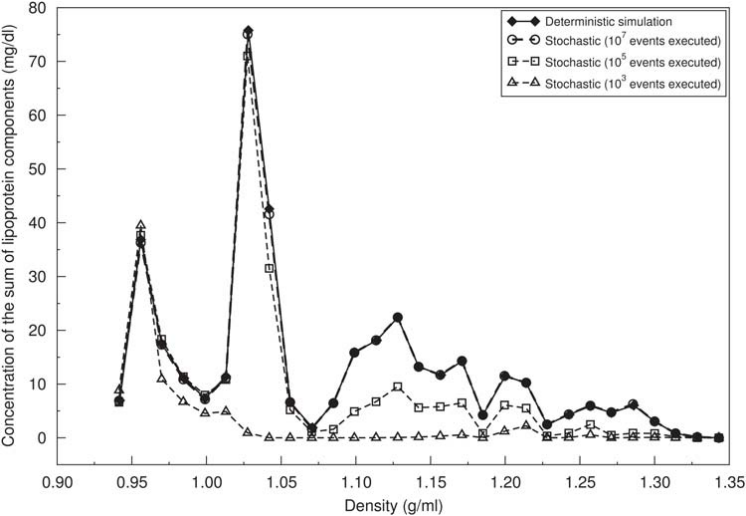
Stochastic versus deterministic simulation. Density distributions of the concentration of the sum of lipoprotein components (mg/dl) obtained by using the Gillespie algorithm with different numbers of simulation steps (events) and by the iterative solution of the deterministic equation system. The density space (0.93–1.35 g/ml) was subdivided into 30 equally sized intervals.

One problem, however, with the Gillespie algorithm is to conclude from the stochastic trajectories at which time point of the simulation the true stationary regime has been reached and a representative sampling of the state space has been accomplished. To test whether the criteria used to assess stationarity work well we have compared the concentrations calculated for one and the same set of kinetic parameters with both simulation variants, the Gillespie algorithm and the deterministic equation system (Figure 2).

To keep the deterministic model numerically tractable we fixed the package sizes for cholesterol (C) and triglyceride (T) to two molecules (A-particles) and 100 and 250 molecules (B-particles), respectively. We also restricted the maximal number of molecules of the components A, B, F, C and T to (4, 0, 5, 100, 40) in an A-particle and to (0, 1, 5, 5000, 10000) in a B-particle. With the package sizes given above, the possible C and T content of an A-particle complex decomposes into 50 and 20 packages; for B-particles they are 50 and 40 packages, respectively.

As the number of all components, with exception of A and B, can become zero, the total number of different lipoprotein complexes in this example is given by 4·(5+1)·(50+1)·(40+1) for A-particles plus 4·(5+1)·(50+1)·(40+1) for B-particles = 38,250, spanning a density range between 0.92 and 1.40 g/ml. Arbitrary values of the kinetic parameters (not shown) were chosen and the stationary concentration of lipoprotein complexes was computed using either Gillespie's algorithm or iterating the fix-point equation (Equation 6).

In order to compare the two solutions we subdivided the total density range covered by the 38,250 lipoprotein complexes into 30 intervals and calculated the occupancy of these density intervals by cumulating the calculated concentrations of the corresponding lipoprotein complexes. As shown in Figure 2, with increasing number of time steps used in the Gillespie simulation the stochastic solution of the Master equation converges toward the numerical solution of the deterministic model.

The striking advantage of performing stochastic simulations of the Master equation by means of Gillespie's algorithm is that increasing the number of lipoprotein components (e.g. by including cholesterol ester) or using smaller package sizes results only in a moderate increase of computing times because this algorithm per se only deals with such lipoprotein complexes that occur with significant concentrations. In contrast, the deterministic model has to deal with all possible complexes despite the fact that most of them never reach discernible concentrations. The following results were obtained with the stochastic simulation algorithm using the much smaller package size of 20 molecules for C and T in B-particles.

### Calculation of Lipoprotein Density Profiles in Healthy Subjects

In the experiment, main density classes of lipoproteins (VLDL, IDL, LDL, HDL) and sub-fractions of LDL and HDL were isolated from blood plasma. The LDL class was separated into six density sub-fractions, which are grouped into the commonly named large buoyant (lb; LDL1/2), intermediate dense (id; LDL3/4), and small-dense LDL (sd; LDL5/6). The HDL class was subdivided into sub-fractions of HDL2b, HDL2a, and HDL3. In the simulation, stationary lipoprotein distributions were computed by using Gillespie's stochastic simulation algorithm as outlined in the Materials and Methods section. Simulation starts in a system state that is lipoprotein-free. Executing approximately five million reactions (one per time step), a steady state was reached, i.e. the average number of lipoproteins and their composition in the systems remained constant. An additional 10 million executions sampled the stationary distribution of individual lipoprotein complexes. We then calculated lipoprotein density profiles as they are typically determined in clinical investigations by assigning each of the lipoprotein complex according to its concentration to one of the experimentally defined density classes.

The set of model parameters that entail best agreement between the computed lipoprotein profile and the experimental data (see Table 2) was determined as follows. To keep the number of lipoprotein complexes in the simulation tractable, we scaled the system with an appropriate volume factor yielding a reaction volume of one tenth femto-liter. Parameter values for the synthesis of A- and B-particles were taken from [9] and [15], respectively, and fixed during parameter optimization. Numerical values of all other model parameters were obtained by minimizing the distance between simulated and clinically measured lipoprotein profiles (see Materials and Methods). They are listed in Table 3.

**Table 2:** Experimental lipoprotein composition data.

Lp fraction	Density (g/ml)		Concentration, mg/dl (±SD) (*n* = 11)						
	Min	Max	Total	A	B	F	C	T	P
Plasma	0.950	1.400	755.8 (102.6)	122.3 (21.7)	68.3 (17.3)	20.87 (3.8)	164.9 (30.1)	109.9 (44.6)	181.9 (23.0)
VLDL	0.950	1.006	120.9 (70.9)	0.0	5.1 (2.1)	7.12 (3.8)	17.1 (9.3)	69.5 (42.8)	22.0 (11.9)
IDL	1.006	1.019	33.4 (10.4)	0.0	3.7 (1.7)	0.54 (0.2)	8.4 (5.1)	12.1 (2.6)	8.6 (3.4)
LDL	1.019	1.063	217.34 (56.1)	0.0	47.5 (12.7)	0.45 (0.4)	89.6 (24.4)	19.7 (5.6)	60.1 (14.6)
LDL1	1.019	1.031	41.8 (12.5)	0.0	7.8 (2.2)	0.05 (0.1)	17.0 (5.9)	5.1 (1.5)	11.8 (3.5)
LDL2	1.031	1.034	30.4 (8.7)	0.0	6.2 (1.7)	0.0	13.0 (4.3)	2.5 (0.7)	8.6 (2.6)
LDL3	1.034	1.037	33.4 (10.0)	0.0	7.3 (2.2)	0.0	14.2 (4.3)	2.6 (1.1)	9.4 (2.7)
LDL4	1.037	1.040	39.7 (17.3)	0.0	9.2 (4.1)	0.0	17.0 (7.5)	2.8 (1.5)	10.8 (4.5)
LDL5	1.040	1.044	36.9 (16.8)	0.0	8.9 (4.1)	0.0	15.5 (7.2)	2.5 (1.2)	10.0 (4.5)
LDL6	1.044	1.063	33.4 (11.6)	0.0	8.4 (3.1)	0.34 (0.2)	12.9 (4.6)	3.0 (1.1)	8.8 (3.0)
HDL	1.063	1.400	216.1 (50.5)	89.4 (19.9)	0.0	5.12 (2.0)	41.8 (10.9)	10.5 (2.0 )	64.8 (18.3)
HDL2	1.063	1.125	96.13 (33.1)	34.9 (12.0)	0.0	0.38 (0.5)	21.61 (8.0)	5.63 (1.0)	33.80 (12.9)
HDL2b	1.063	1.100	31.1 (16.8)	9.4 (5.5)	0.0	0.38 (0.4)	8.1 (4.3)	2.0 (0.4)	11.3 (6.5)
HDL2a	1.100	1.125	65.1 (18.6)	25.5 (7.3)	0.0	0.0	13.5 (4.2)	3.6 (0.8)	22.5 (7.2)
HDL3	1.125	1.210	84.2 (17.3)	42.8 (9.1)	0.0	0.48 (0.4)	14.4 (3.0)	3.6 (1.1)	23.2 (5.4)
pre*β*-HDL^a^	1.210	1.400	40.8 (20.0)	30.6 (8.0)	0.0	2.57 (1.5)	0.0	0.0	29.7 (1.3)

The data are averaged values from 11 randomly selected normolipidemic subjects.

a The density fraction of so-called pre*β*-HDL (1.210–1.400 g/ml) was not directly measured in the experiment. We therefore assumed and calculated this fraction as the difference between total plasma and total HDL values for apoA-I, cholesterol, and phospholipids.

**Table 3:** Model parameter values.

Rate Constants (*cμ*)		Unit	Model Value	Exp Value	Reference
A-particle	*c_createA_*	mmol/l·day^−1^	8.0e-3	8.4e-3 to 9.2e-3	[9],[15],[53]
	*c_destroyA_*	day^−1^	0.21	0.20	[9],[15],[53],[54]
	*c_influx_*	mmol/l·day^−1^	1.1e-3	2.5e-3	[55]
	*c_effluxA_*	day^−1^	0.01	0.312	[54]
	*c_exchangeCA_*	day^−1^	397.1	110.1	[56] ^a^
	*c_exchangeTA_*	day^−1^	0.65	-	
	*c_transferA_*	day^−1^	2.0e-4	5.3e-5	[53] ^g^
	*c_uptakeA_*	day^−1^	0.02	0.14	[53] ^h^
	*c_transferFA_*	day^−1^	9.4e-4	7.6e-3	[16] ^c^
	*c_uptakeFA_*	day^−1^	1.9e-3	3.9e-3	[16] ^d^
	*c_hydrolyzeA_*	day^−1^	5.6	27.72	[57]
B-particle	*c_createB_*	mmol/l·day^−1^	1.0e-3	1.0e-3, 1.4e-3	[15],[58]
	*c_destroyB_*	day^−1^	1.31	0.5 - 5.5	[15]
				0.4 - 6.9	[58]
	*c_effluxB_*	day^−1^	0.5	-	
	*c_exchangeCB_*	day^−1^	1.7	-	
	*c_exchangeTB1_*	day^−1^	887.75	1.2	[56] ^b^
	*c_exchangeTB2_*	day^−1^	55.7	-	
	*c_transferFB_*	day^−1^	2.0e-3	1.6e-3	[16] ^e^
	*c_uptakeFB_*	day^−1^	3.5e-3	0.061	[16] ^f^
	*c_hydrolyzeB_*	day^−1^	8.3	7.52	[54]

Comparison of estimated model parameter values with measured rate constants found in the literature.

**a–h:** Indexes; see explanations in Dataset S3. For ^a^ and ^b^, it might be more useful to compare the flux values (total transfer activities, mg/dl per day), because the literature substrate concentrations vary from that in our simulation. HDL-CE is approximately one third and VLDL-TG is approximately double of that in our simulation. In both cases, the CETP mass is much less even.

a 72.13 vs. 110.52 mg/dl · day^−1^.

b 297.45 vs. 15.87 mg/dl · day^−1^.

The estimated parameter values are in most cases in a reasonable agreement with experimentally determined values taking into account the difficulties to extract rate constants of elementary processes from kinetic measurements settled on compartment analysis. The underlying reaction mechanism can either be monomolecular or bimolecular which is important to know while comparing the stochastic rate constants with rate constants obtained from tracer kinetic studies. A detailed comparison of calculated and measured rate constants is given in Dataset S3.

**Figure 3:**
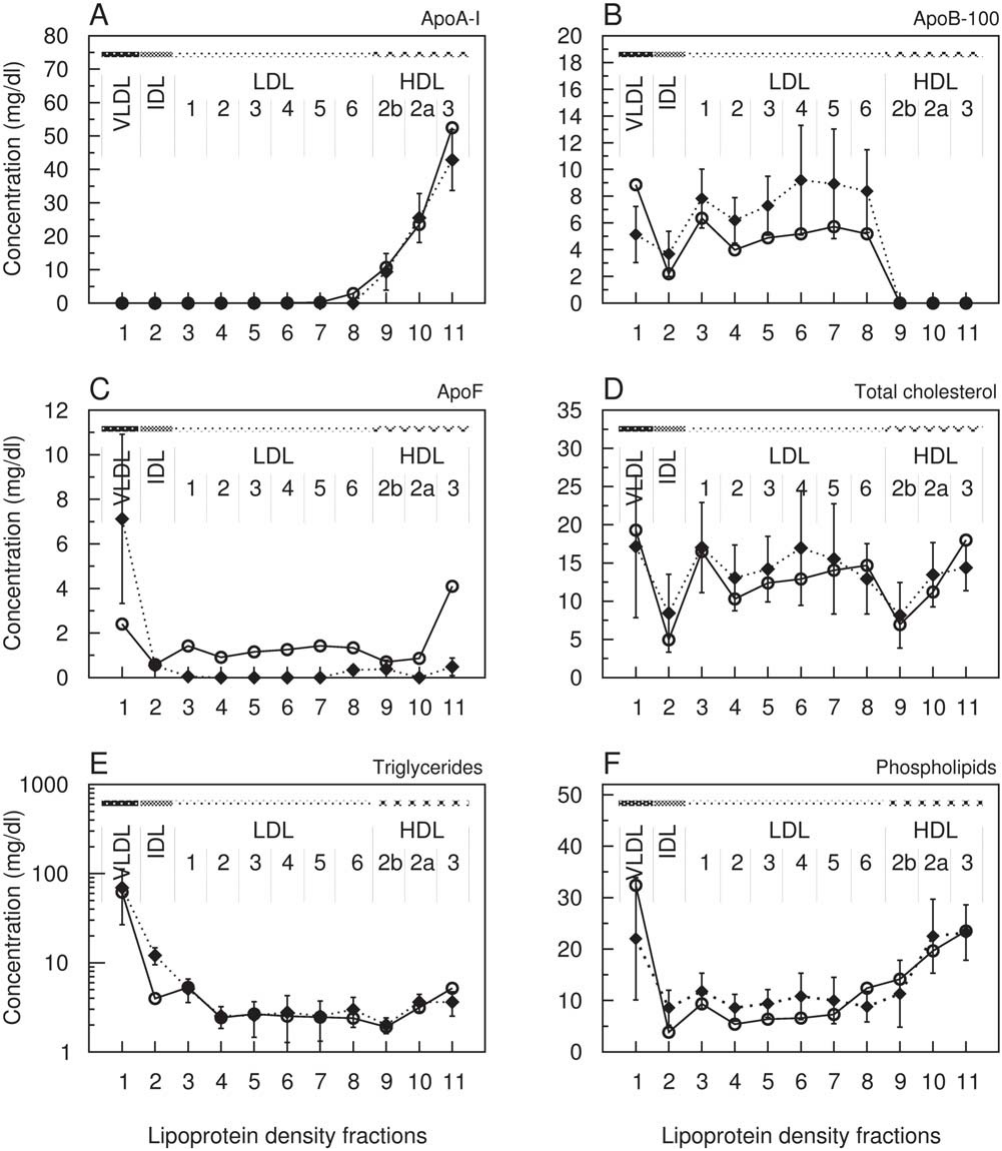
Simulated versus clinically measured distribution of lipoprotein components over main density classes including sub-fractions of LDL1-6, HDL2b, HDL2a and HDL3. *x*-axis: number of lipoprotein density fraction; *y*-axis: simulated (circles) versus clinically measured (rectangles) concentration values in mg/dl of Apolipoprotein A–I (A), Apolipoprotein B-100 (B), Sum of further apolipoproteins (C), Total cholesterol (D), Triglycerides (E, logarithm) and Phospholipids (F). The error bars show the standard deviation of the experimental values.

As shown in Figure 3, the calculated lipoprotein density profiles for each of the lipoprotein components by using the parameter values given in Table 3 are, to a large part, in a remarkable agreement with the clinical data. However, with respect to the distribution of apolipoprotein B (Figure 3B) and of triglycerides (Figure 3E) some discrepancies remain.

The total amount of 41.8±0.45 mg/dl of component B predicted by the model is lower than the mean value of 56.6 mg/dl determined experimentally for apoB-100, but within the expected interval (±21.4 mg/dl). Compared with experimental values the calculated concentration of apoB-100 is higher in the VLDL sub-fraction but lower in IDL and all LDL sub-fractions. This might be accounted by the simplifications made in our model for the kinetics of triglyceride removal from B-particles because regulatory influences of apolipoproteins C and E are ignored. Likewise, the simplified kinetics of triglyceride removal from B-particles might also explain the too low triglyceride content predicted for the IDL sub-fraction since the high rate of triglyceride hydrolysis obtained by the parameter optimization procedure yields a rapid delipidation of newly synthesized B-particles. The simplification to assume a definite initial composition of newly synthesized lipoproteins in the model might be another reason for the remaining discrepancy in the distribution of apoB-100.

The calculated distribution of model component F was compared with the clinically measured concentration values of the C apolipoproteins I–III and apoE. However, experimental data for component F are questionable, as only about one-half of the total plasma concentration of apoC and apoE (20.87±3.8 mg/dl) is associated with lipoprotein complexes. The other half reflects a free apolipoprotein pool in plasma, whose value is about 10-fold higher than 1.2 mg/dl reported by [16]. This might result from experimental difficulties, because it is well documented that apoE may dissociate from the surface of apoB-containing particles during prolonged ultracentrifugation [17],[18]. This may account for the fact that our model predicts higher levels of component F in almost all lipoprotein density classes as compared to the available experimental data (Figure 3C). In fact, the calculated distribution of F agrees much better with the experimental total plasma concentration (19.0 mg/dl) as well as with the concentration observed for the free plasma pool (1.2 mg/dl) by Batal et al.

#### Initial composition analysis

The initial composition of A- and B-particles was kept constant during simulation (Table 1). However, depending on a variety of factors, including nutrition or cellular and regulatory processes, the liver generates a certain lipoprotein spectrum of different compositions. To analyze how the lipoprotein distribution in the blood plasma changes in response to different initial compositions, the molecule numbers of the lipoprotein components F, C, and T of B-particles were randomized from a normal distribution by taking the reference composition as the mean value. A total of 100 different initial compositions were analyzed in independent simulation runs. The compositions obtained by randomization provide ranges for F, C, and T between 0–18, 760–2,934, and 6,654–14,784 molecules, respectively. The variation of each of the random compositions relative to the reference composition was quantified using the euclidean distance.

**Figure 4:**
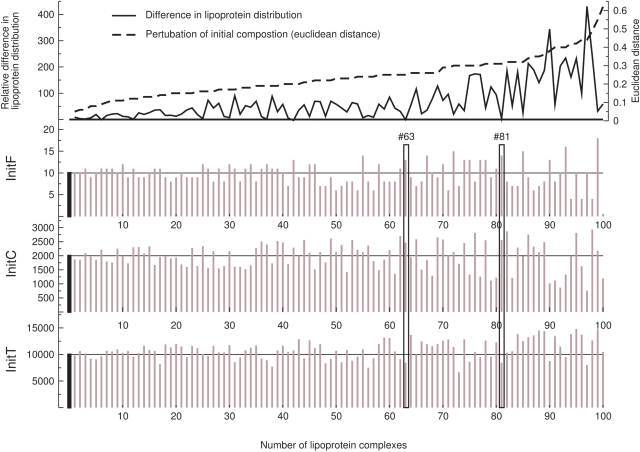
Variation in the initial composition of B-particles. The initial composition of B-particles, i.e. the molecule numbers of component F, C and T, was randomized. The filled black bars (x = 0) mark the default B-particle initial composition (initF = 10, initC = 2000, initT = 10000). A total of 100 different compositions (brown bars) were analyzed by independent simulation runs. The graphs are sorted by the euclidean distance (topmost sub-graph, black dashed line). The change in the error measure (distance between calculated and experimental lipoprotein distributions) relative to the value obtained for the default composition is shown in the topmost sub-graph (black continous line).

Figure 4 illustrates the overall tendency, the more different the composition from the reference value (increasing euclidean distance) the larger the distance between the calculated and experimental lipoprotein distributions. However, a number of compositions considerably deviating from the default one fit comparably well or even improve the agreement with the experimental data (see LP composition #81 and #63, respectively). In our calculations, this predominantly pertains to compositions that are mainly increased in the amount of component F and C. The results suggest that certain variability in the initial composition can be partially compensated by the kinetic processes in the LP metabolism. Or, the other way around, specific inter-individual variations in the liver status are not necessarily reflected in altered distributions of the main lipoprotein classes in the blood.

### hrDS

Characterizing the distribution of lipoproteins in the blood plasma by quantifying their abundance in a limited number of main density classes such as chylomicrons, VLDL, IDL, LDL, and HDL (the classical density profile) appears feasible as long as the distribution of lipoprotein components within these classes is sufficiently smooth. That means, any alteration in the kinetic properties of the underlying elementary processes ultimately gives rise to changes in the relative occupation of these density classes. On the other hand, alterations in the kinetic processes may not necessarily lead to visible changes in the average value of a density class, while the concentration and composition of individual lipoproteins within the class may vary significantly.

To reveal the heterogeneity of the lipoprotein distribution within the main density classes experimentally used, the width of each was decomposed into five equally sized sub-intervals. Subsequently, we quantified the calculated amount of lipoprotein components in these narrow density classes, for which we introduce the name hrDS. Since the main density classes exhibit differing density interval sizes, we normalized the distribution within each density class to its interval size.

**Figure 5:**
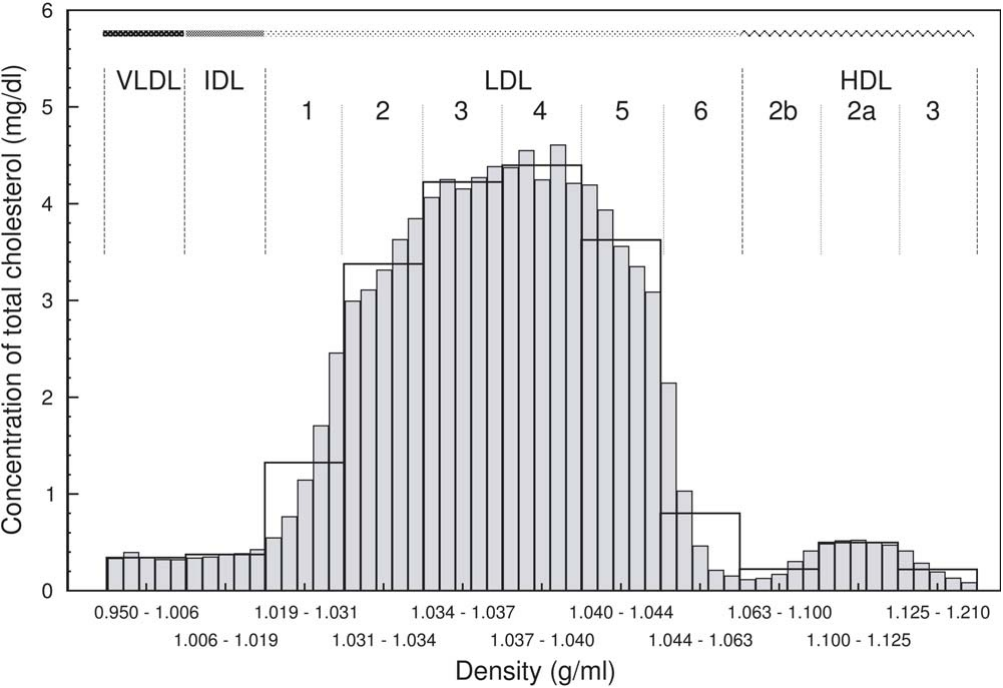
High-resolution distribution of total cholesterol within main density classes including sub-fractions of LDL1-6, HDL2b, HDL2a, and HDL3. Each density class was further decomposed into 5 equally sized sub-fractions, called hrDS (grey bars). *x*-axis: density in g/ml; *y*-axis: concentration of total cholesterol in mg/dl normalized to the density interval size.

As an example, Figure 5 shows the distribution of cholesterol across the hrDS, which allows to quantify the contribution of the hrDS to the average concentration of the main classes. Most significant intra-class variation appear in the ascending (LDL1, LDL2 and HDL2b) and descending (LDL5, LDL6 and HDL3) part of the overall distribution. For example, in LDL6 (density d = 1.044–1.063 g/ml), the calculated mean concentration of cholesterol amounts to 15.1±0.1 mg/dl (normalized value of 0.795 with LDL6 interval size of 19). The five hrDS named LDL6(I), (II), (III), (IV), and (V) relatively contribute with 53.1%, 25.7%, 11.7%, 5.5%, and 4.0% to the average cholesterol concentrations, respectively. According to this finding, more than one-half of the cholesterol content in LDL6 is contributed by lipoprotein complexes with densities in the narrow range of 1.044–1.0478 g/ml. Within the other classes the hrDS are nearly equally distributed.

**Figure 6:**
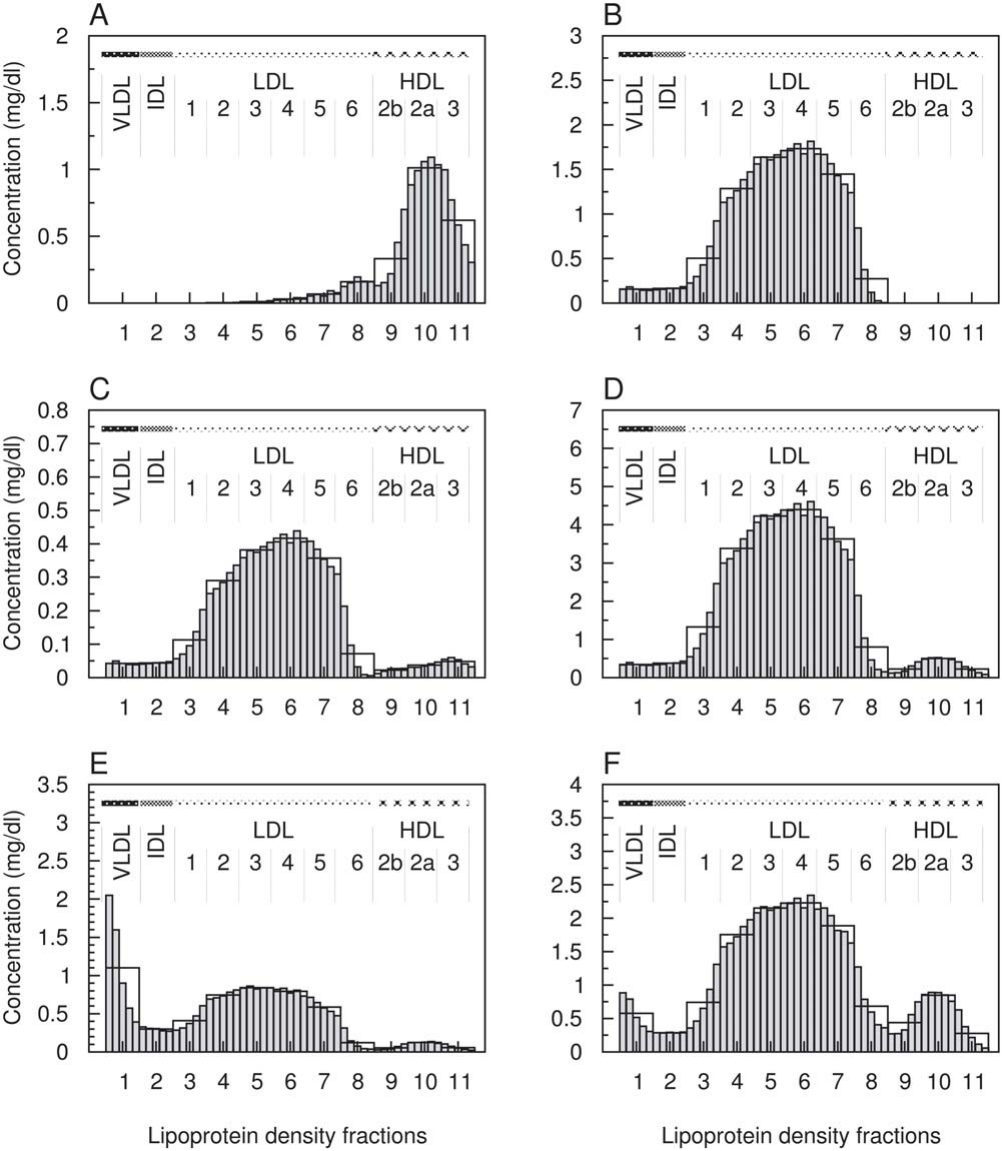
High-resolution distribution of lipoprotein components within main density classes including sub-fractions of LDL1-6, HDL2b, HDL2a, and HDL3. Each density class was further decomposed into five equally sized sub-fractions, called hrDS (grey bars). *x*-axis: number of lipoprotein density subfraction; *y*-axis: concentration of apolipoprotein A–I (A), apolipoprotein B-100 (B), sum of further apolipoproteins (C), total cholesterol (D), triglycerides (E) and phospholipids (F) in mg/dl normalized to the density interval size.

The specific intra-class variations of the main density classes show similar patterns for all the other lipoprotein components (Figure 6). However, some intra-class distributions vary for different components. For example, within VLDL triglycerides and phospholipids show monotonically decreasing instead of a nearly equal distribution (Figure 6E, F).

One may hypothesize that the amount and distribution of lipoprotein sub-populations differ between individual healthy subjects or even in pathological conditions due to inter-individual variations. To check whether the knowledge of the intra-class distribution provides additional and valuable information, we moderately varied each of the kinetic parameters by ±10% of the reference value.

**Figure 7:**
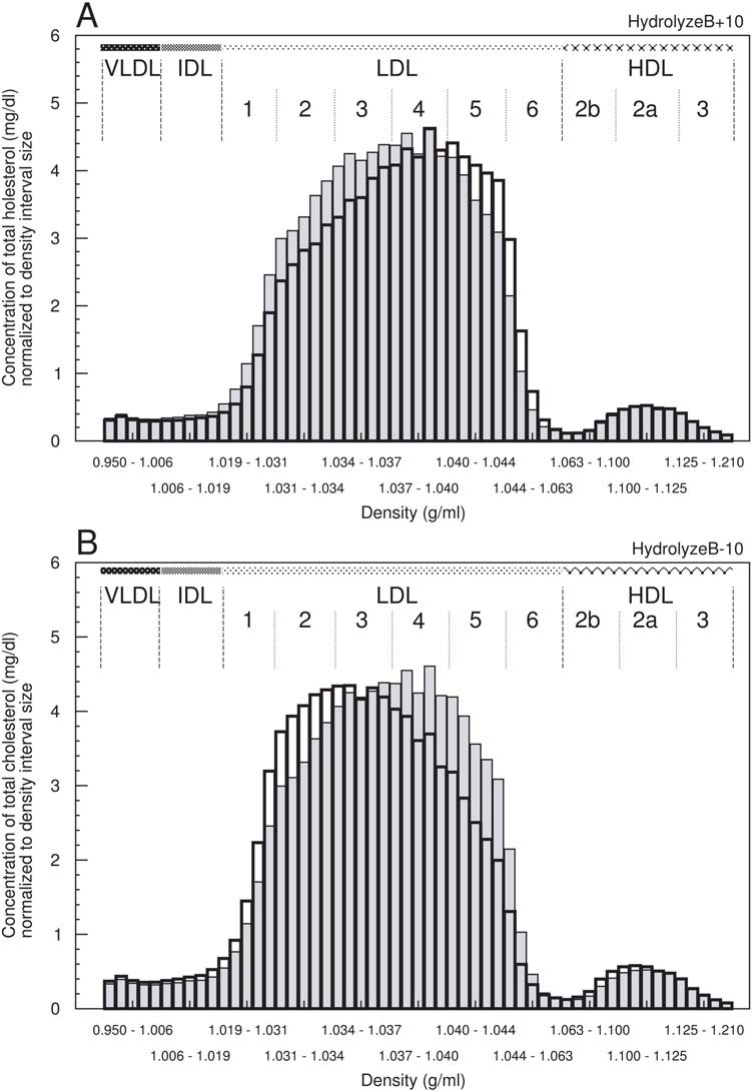
Variation in the distribution of hrDS cholesterol at moderately altered parameter values. Alteration of the normal hrDS cholesterol distribution (grey bars) by (A) increasing and (B) decreasing the parameter value of the *HydrolyzeB* process by 10% of its normal value (black bars). *x*-axis: metrical density intervals in g/ml; *y*-axis: concentration of total cholesterol in mg/dl normalized to the density interval size.

The results indicate that, for example, marginal alterations in the delipidation process of B-particles (*HydrolyzeB*) shifts the high resolution distribution within a major density class either to lower or higher densities (Figure 7), while the concentration value of the major classes (e.g., LDL) remains nearly unchanged. Similar results were obtained for the selective cholesteryl ester uptake from B-particles (*EffluxB*) and the amount of CETP available (data not shown).

### Simulated Pathological States

**Figure 8:**
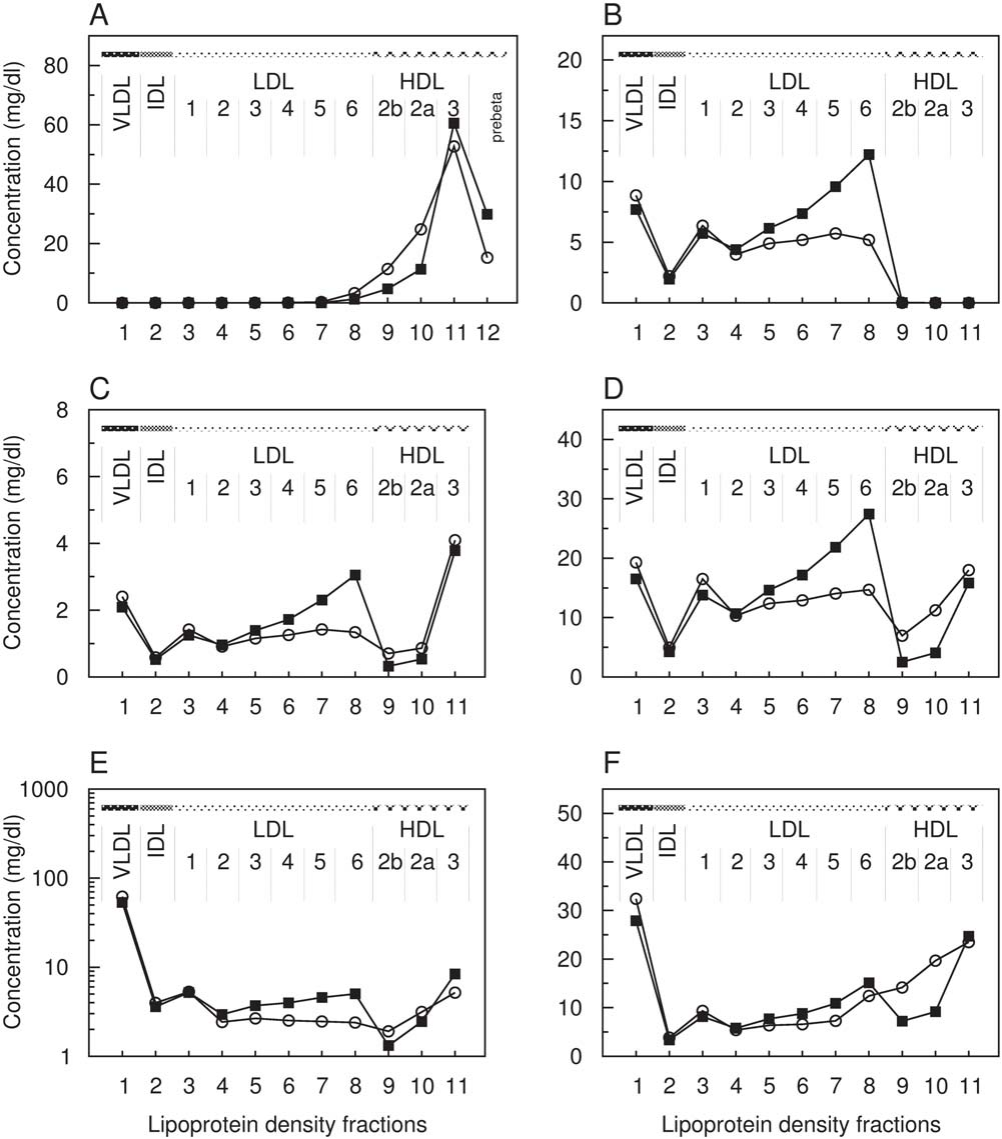
Calculated pathological distribution compared with calculated normal data for LDL receptor deficiency. Distributions of the lipoprotein components in the main density classes including sub-fractions of LDL1-6, HDL2b, HDL2a and HDL3. *x*-axis: number of lipoprotein density fractions; *y*-axis: calculated pathological (squares) and calculated normal (circles) concentration values of apolipoprotein A–I (A), apolipoprotein B-100 (B), sum of further apolipoproteins (C), total cholesterol (D), triglycerides ([E], logarithm) and phospholipids (F) in mg/dl.

To check the predictive capacity of our model we simulated the impact of disorders in the kinetic properties of the LDL-receptor (LDLR; P01130, ENSG00000130164), the lipoprotein lipase (LPL) and ATP-binding cassette A1 (ABCA1; O95477, ENSG00000165029) on the stationary density distribution of lipoproteins (Figures 8–10).

**Figure 9 pcbi-1000079-g009:**
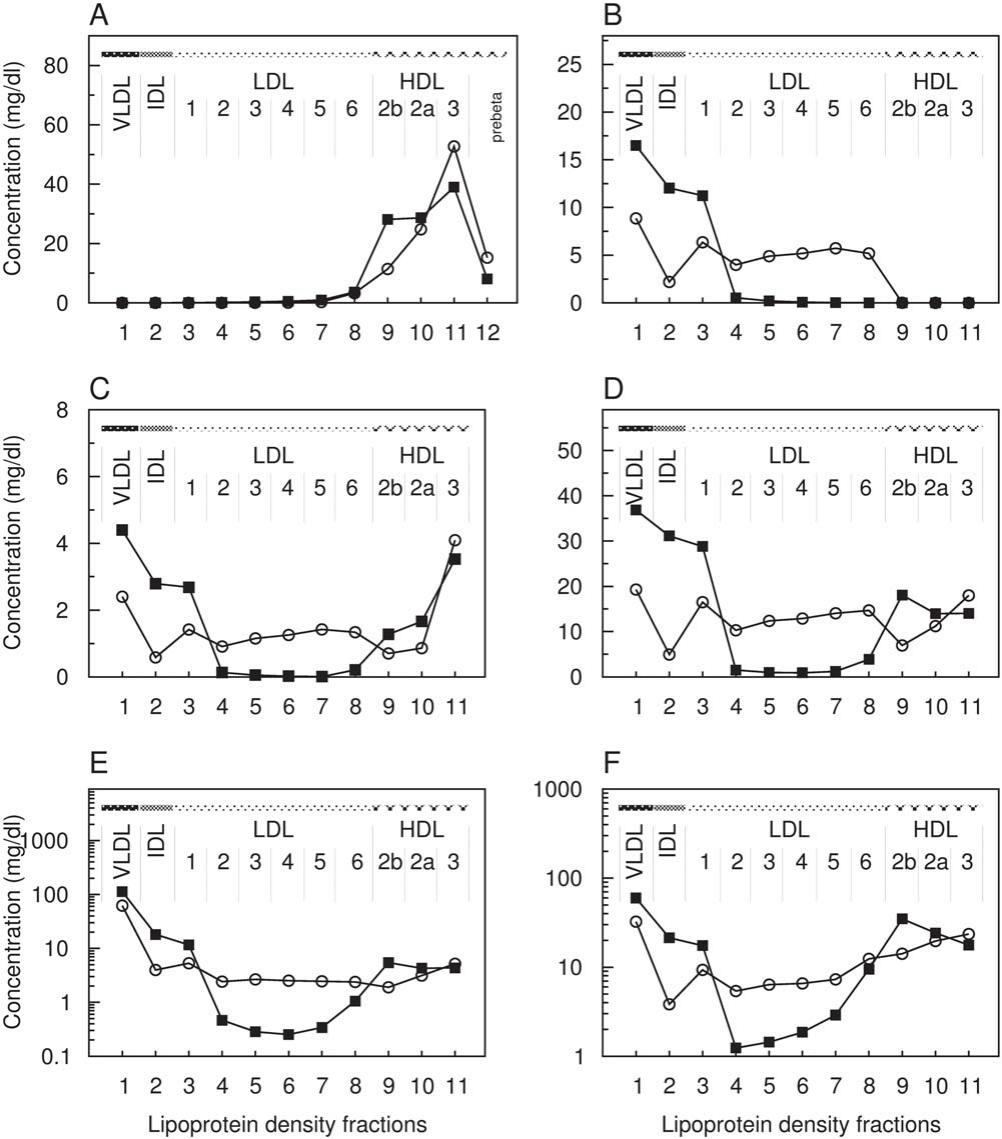
Calculated pathological distribution compared with calculated normal data for LPL deficiency. Distributions of the lipoprotein components in the main density classes including sub-fractions of LDL1-6, HDL2b, HDL2a, and HDL3. *x*-axis: number of lipoprotein density fractions; *y*-axis: calculated pathological (squares) and calculated normal (circles) concentration values of apolipoprotein A-I (A), apolipoprotein B-100 (B), sum of further apolipoproteins (C), total cholesterol (D), triglycerides ([E], logarithm), and phospholipids (F, logarithm) in mg/dl.

**Figure 10:**
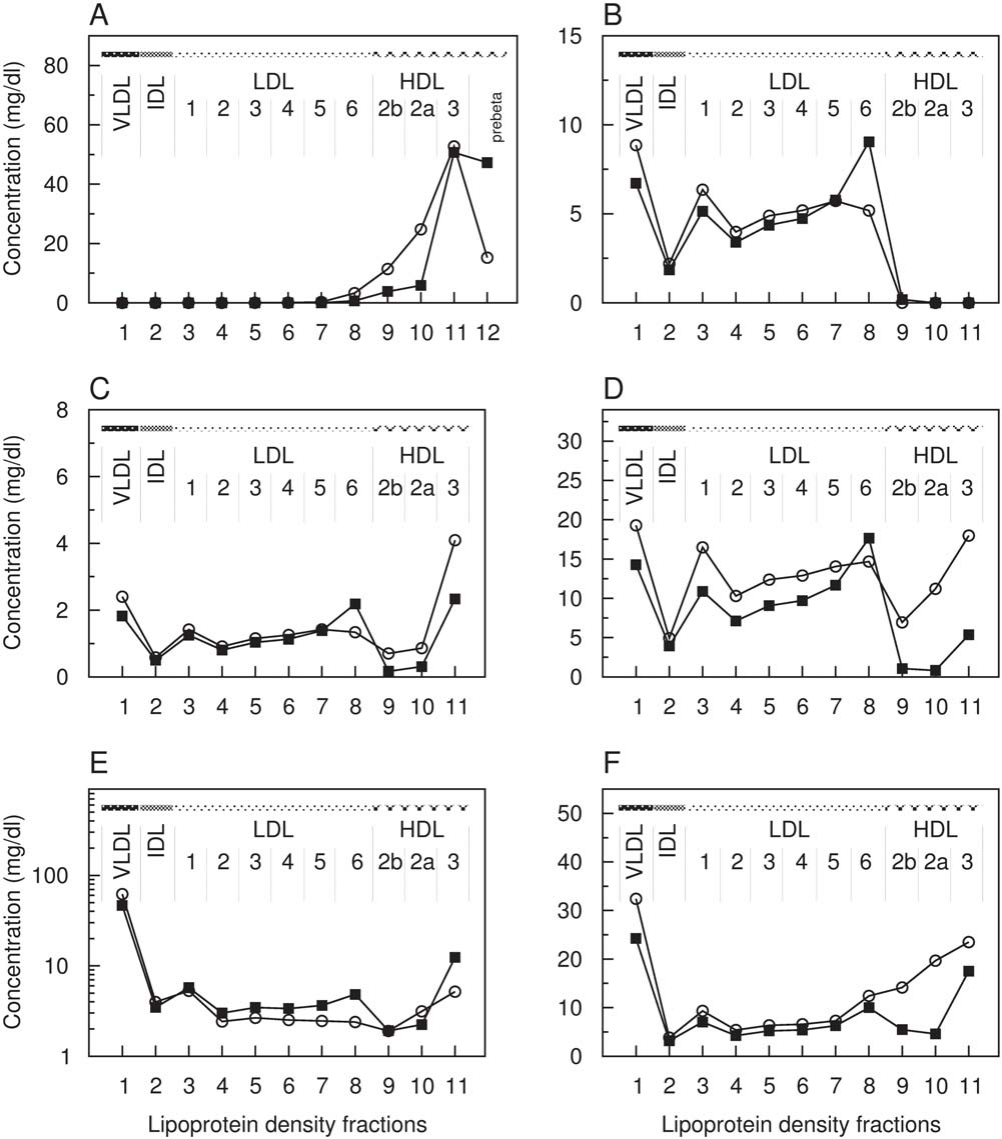
Calculated pathological distribution compared with calculated normal data for ABCA1 deficiency. Distributions of the lipoprotein components in the main density classes, including sub-fractions of LDL1-6, HDL2b, HDL2a and HDL3. *x*-axis: number of lipoprotein density fractions; *y*-axis: calculated pathological (squares) and calculated normal (circles) concentration values of of apolipoprotein A–I (A), apolipoprotein B-100 (B), sum of further apolipoproteins (C), total cholesterol (D), triglycerides ([E], logarithm), and phospholipids (F) in mg/dl.

**Familial Hypercholesterolemia (FH)** is an autosomal hereditary disease caused by a dysfunction of the LDLR. We simulated a reduced LDLR activity by decreasing the parameter for the process *DestroyB* to 75% of its normal value. The calculated lipoprotein distribution exhibits an increased concentration of LDL cholesterol at nearly unchanged cholesterol levels of VLDL and IDL (Figure 8D). It is suggesting that this arises from lowering the receptor-mediated uptake while maintaining a sufficient apoB-synthesis leading likewise to elevated LDL-apoB levels (Figure 8B).

Within LDL, the sub-fractions LDL1-6 behave differently. As compared to normolipidemic cholesterol values, we observe moderately increased levels of idLDL-C (25.2 vs. 31.8 mg/dl, +26%) and to a higher extent of sdLDL-C (28.7 vs. 49.2 mg/dl, +71%) whereas lbLDL-C (26.8 vs. 24.4 mg/dl) remains nearly unchanged. These results coincides with findings of low LDL-cholesterol to LDL-apoB ratios in carriers of the FH-Keuruu mutation (Asp235→Glu) suggesting that LDL particles are small and dense [19]. The LDL subfraction pattern is also similar to findings by März et al. in a patient with FDB (Familial defective apolipoproteinB-100) who has a mutation in codon 3500 of the apoB gene substituting glutamine for arginine [20].

Regarding the distribution of A-particles (equivalent to HDL), the model predicts a moderate decrease in HDL cholesterol as compared to the normolipidemic profile (Figure 8D). This is in good agreement with the reduced overall HDL cholesterol level observed in heterozygous FH patients [7].

However, an impaired interaction between B-particles, such as LDL, and the receptor may be due to several reasons. Either there is indeed a defect in the receptor itself (FH) due to mutations that cause a reduced expression or binding activity, or the ligand (potentially apolipoprotein B-100) carries a mutation (FDB). Since the uptake process is determined by solely one parameter in the present model, we cannot yet discriminate between different molecular determinants.

**Hypertriglyceridemia** is characterized by elevated levels of plasma triglycerides caused, among others, by deficiencies of the lipoprotein lipase (LPL), the key enzyme in the catabolism of triglyceride-rich lipoproteins by removing (hydrolyzing) triglycerides. We simulated the consequences of an impaired LPL activity by lowering the parameter value for the process *HydrolyzeB* to one-half of the original value.

The calculated lipoprotein distributions display markedly elevated levels of triglycerides as well as cholesterol, predominantly of VLDL, IDL and early LDL (Figure 9D and 9E). The total plasma concentration of triglycerides (159.5 vs. 95.2 mg/dl) is about 67.4% increased as compared to the simulated normolipidemic profile. According to our calculations, the total concentration of LDL cholesterol is only marginally affected (152 vs. 145 mg/dl), whereas substantial alterations in the cholesterol level of individual LDL subfractions are predicted. lbLDL (LDL1+2) is considerably increased. As compared to normolipidemic LDL cholesterol values, we obtain a strong reduction in idLDL (LDL3+4, 25.2 mg/dl vs. 1.8 mg/dl, −92%) and to a lower degree of sdLDL (LDL5+6, 28.7 mg/dl vs. 5.1 mg/dl, −82%). Elevated levels of sdLDL implicated with mild to moderate hypertriglyceridemia cannot be found in the model simulations [21]. Likewise, reduced HDL cholesterol are not observed as reported by Babirak et al. for the phenotype of heterozygous LPL deficiency [22]. In contrast, the calculated distribution shows increased HDL cholesterol levels of HDL2b while in HDL2a and HDL3 no discernible changes occur.

The calculated distributions might be mechanistically explained as follows. Due to the decreased hydrolysis parameter, during simulation, the *HydrolyzeB* process is executed about 1.2-fold less leading to the accumulation of B-particles enriched in triglycerides. Elevated triglyceride levels, in turn, promote the transfer of triglycerides to A-particles mediated by the CETP (1.4-fold higher frequency of the processes ExchangeT_B_ and ExchangeT_B_). Likewise, the more CETP is loaded with triglycerides the less lipid-unloaded CETP is capable to transport cholesterol back from A-particles to B-particles. Subsequently, lipoprotein complexes in the density range of HDL become enriched in cholesterol and triglycerides. A 1.4-fold higher frequency is also observed for the *HydrolyzeA* process implicating a higher HL activity, the enzyme that catalyzes the hydrolysis of triglycerides from small B-particles and A-particles. That may explain the low triglyceride content in all LDL sub-fractions.

**Hypoalphalipoproteinemia** is a rare human metabolic disorder characterized by a severe decrease in HDL cholesterol and apoA-I levels. For example, Tangier Disease (TD) is assumed to be caused by defects in both alleles of the ABCA1 (ATP-binding cassette A1) transporter gene [23], the key mediator of the reverse cholesterol transport by transferring cholesterol and phospholipids from peripheral cells to acceptor lipoproteins in the plasma. A heterozygous ABCA1 defect correspond to the disorder known as familial hypoalphalipoproteinemia (FHA).

We simulated an impaired activity of ABCA1 by reducing the rate constant for the cholesterol uptake into A-particles (process *Influx*) to 50% of its normal value. Compared with simulated normolipidemic values, the model predicts low plasma cholesterol concentrations (144.5 mg/dl vs. 95.47 mg/dl, −34%). As reported for FHA, a remarkable reduction of cholesterol levels appears in all HDL fractions (Figure 10D). HDL cholesterol accounts for only ∼10 mg/dl at nearly normal total plasma triglyceride levels (95.2 vs. 96.8 mg/dl). As a consequence, considerable levels of apoA-I occur in the density range d>1.21 g/ml which argues for the accumulation of pre-β–migrating lipoproteins (Figure 10A). Our model simulation predicts further a marginal reduction in apoA-I within the HDL3 fraction, while in HDL2b and HDL2a apoA-I is selectively depleted. As comparable results from clinical studies, a predominance of HDL particles being poor in cholesterol but enriched in apoA-I in patients with heterozygous TD [24], [25].

The distributions of apoB, apoF, cholesterol and triglycerides of B-particles display a shift to lipoproteins in the density range of LDL6 (Figure 10B–10E). Analyzing the stochastic trajectories of our model simulations shows that the *Influx* process occurred approximately 2-fold less frequent. The reduced uptake of cholesterol from the periphery to A-particles leads to a diminished cholesterol transfer to B-particles and to less CETP molecules loaded with cholesterol. This forces the rate for the back transfer of triglycerides from triglyceride-rich B-particles to A-particles or cholesterol-rich B-particles to increase. Accordingly, the model calculations show reduced concentrations of VLDL triglycerides and increased triglyceride levels in, e.g. HDL3 and LDL6, respectively.

## Discussion

We have developed a novel computational approach toward the calculation of lipoprotein distributions in blood plasma. The basic idea of our concept is to model the dynamics of individual lipoproteins instead of predefined density classes. This enables us to include in an adequate manner the elementary reactions involved in lipoprotein metabolism. A further benefit of our approach is to provide a more detailed information on the lipid and protein composition of lipoproteins than possible by using conventional compartment models.

To introduce the method and to deal with a manageable set of unknown kinetic parameters, we present in this work a simplified core model which does not include all biochemical processes involved in lipoprotein metabolism and which uses simplified rate equations of the mass-action type. Therefore, it is obvious that some inconsistencies between calculated and measured distributions of lipoprotein should occur. Nevertheless, even based on this simplified core model we were able to simulate with remarkable accuracy experimentally determined density profiles of lipid and protein components in normal and pathological situations.

### Calculated Versus Clinically Measured Lipoprotein Profiles in Healthy Subjects

As experimental information on the composition of lipoproteins so far is only available for the commonly defined density classes (lipoprotein compartments) the only way to estimate unknown parameters of the model and to compare the computations with the experiment was to condense the calculated profiles of individual lipoprotein complexes into density class profiles. This way, we determined numerical values of model parameters which, to a large degree, are in good agreement with experimental data taken from a larger set of independent kinetic experiments.

Based on this parametrization, we computed lipoprotein density profiles of healthy subjects. The remaining deviations pertain mostly to the distribution of apoB-100, whose calculated concentration is higher in VLDL and lower in the IDL and LDL classes than measured in the blood of normolipidemic patients. Further refinement of the model by including, for example, the regulation of LPL activity by apolipoprotein C-II [26] or the hepatic generation of a broader spectrum of lipoproteins even belonging to the IDL and LDL type [14] will certainly help to overcome this discrepancy. However, discrepancies between model and experiment may at least partially also result from experimental uncertainties.

### Simulated Pathological States

The model was applied to calculate the distribution of lipoproteins of subjects with a defined molecular defect in one of the underlying elementary kinetic processes. Hypercholesterolemia, hypertriglyceridemia and hypoalphalipoproteinemia were exemplarily simulated by modifying the corresponding model parameters.

In all cases, the simulated pathological states could nicely reproduce fundamental clinical characteristics of the selected dyslipidemia. It has to be noted, that, in the present state of the model, we cannot assign the altered lipid phenotype to different molecular determinants of a process. For example, hypercholesterolemia may caused by a reduced binding constant of apoB or a diminished expression of the apoB-binding LDL receptor. Refining the kinetic description of the processes will be therefore one of the tasks in future work and might allow to address defects in different genes to a process.

### Lipid Values in High Resolution

Various studies have shown that individual lipoprotein sub-populations exhibit differing metabolic behavior. First evidence for the existence of discrete LDL sub-populations has been reported by Krauss and Burke [27]. More recently, also HDL has been found to be especially complex with at least 5 and perhaps as many as 12 or more subclasses [28] showing differing metabolic behavior [29] and redistribution in pathological conditions [25].

Nevertheless, experimental separation and analysis is an elaborate, time-consuming and expensive venture and not yet worthwhile for routine measurements. Experimental methods established for lipid fractionation include gradient density ultracentrifugation [30]–[32], non-denaturing polyacrylamide gradient gel electrophoresis (GGE) [33], nuclear magnetic resonance (NMR) spectroscopy [34],[35] and high performance liquid chromatography (HPLC) [36],[37], each with particular assets and drawbacks [38]. GGE and NMR spectroscopy in particular are capable of measuring both lipoprotein particle numbers (LDL-P) and size. In fact, the cholesterol content per particle exhibits large inter-individual variation, and distributions of LDL sub-classes have been shown to vary tremendously among individuals independent of total LDL cholesterol [39], which emphasizes the importance of the concept presented here as the work permits to calculate the distribution of lipoproteins across any narrow density interval of choice and on the basis of the entire spectrum of lipoprotein particles in plasma differing in size, composition and physiological function.

The analysis of high-resolution lipoprotein profiles, therefore, preferentially aim at understanding the reasons for inter-individual variability in subjects of normal or intermediate risk state, but possibly even in distinct pathological conditions. To this end, experimental validation of the predicted high resolution distribution will be essential in future work.

### Model Extensions and Refinements

Based on our findings, we plan to study in a systematic manner how a re-definition of density classes and the combination of lipoprotein component levels determined within these classes may help to define novel diagnostic parameters which sensitively and specifically indicate alterations of the lipoprotein metabolism on the molecular level. However, such model-based optimization of systemic lipid diagnostics requires extensive improvements of the core model presented in this paper. The most relevant extensions and refinements necessary to increase the physiological reliability of the model are as follows: (i) inclusion of apoA-II (P02652, ENSG00000158874) to allow for differentiation between LpA-I and LpA-I:A-II particles to better satisfy the differing metabolic behavior of several HDL sub-populations in normal and pathological conditions [25],[40]; (ii) inclusion of apoB-48 in addition to apoB-100 to model the metabolism of intestinal synthesized chylomicrons even with respect to postprandial hyperlipidemia [41],[42]; (iii) distinguishing between free cholesterol and cholesteryl ester and inclusion of the esterification process of free cholesterol by LCAT [43]; (iv) disaggregation of the model component variable F into apolipoproteins E and C and explicit consideration of the regulatory function of these isoforms (e.g., activating effect of apoC-I [P02654, ENSG00000130208] on LCAT [44], activation of LPL by apoC-II [P02655, ENSG00000213044] [26], influence on the LDL receptor binding by apoE [45], and the apoE-dependent alternative path for peripheral cholesterol [46]); (v) explicit incorporation of the phospholipid exchange mediated by the phospholipid transfer protein (PLTP; P55058, ENSG00000100979) playing a key role in the remodeling of HDL [47],[48]; and (vi) inclusion of other transporters and receptors involved either in the holoparticle uptake or in the uptake of individual lipoprotein components, e.g., SR-B1 (Q8WTV0, ENSG00000073060), ABCG1 (P45844, ENSG00000160179), and ABCG4 (Q9H172, ENSG00000172350) [49],[50].

### Model-Based Clinical Application

The clinical relevance of our modeling approach consists in its capability to infer from the measured lipoprotein profile of a patient potential alterations in one or several of the underlying kinetic processes. Together with other independent information on diet (affecting primarily the composition and amount of VLDL particles generated by the liver) and genetic variations based on SNP analysis of genes related to enzymes of the lipoprotein metabolism this will allow to elucidate the molecular basis of observed abnormal lipoprotein profiles.

It has to be critically noted, however, that there is no unambiguous relationship between the conventionally measured pattern of lipoprotein main classes (VLDL, IDL, LDL, HDL2, HDL3) and the kinetic parameters of the kinetic processes included in the model. In other words, different sets of kinetic parameters may provide one and the same calculated pattern of main classes. One reason is that moderate changes in the kinetic properties of a single process may cause a moderate shift in the lipoprotein distribution that does not significantly affect the average composition of the main classes. This has been demonstrated by comparing our calculated lipoprotein distribution of a normal patient and a virtual patient having a 10% lower or higher activity of his lipoprotein lipase. The absolute amount and lipid composition of the main density classes of both patients are practically identical. The example also illustrates the advantage of high-resolution profiles for making the altered lipoprotein distribution within the main classes visible. A second reason accounting for the above mentioned ambiguity between the lipoprotein density profile and the kinetic parameters of the underlying molecular processes is that simultaneous alterations in more than one parameter may compensate each other with respect to the resulting shape of the LP distribution. An exemplary case was shown in that the initial composition of the VLDL particle leaving the liver was varied and the distance of the associated lipoprotein density profile was computed with respect to the standard profile. Notably, there exist different initial compositions yielding practically identical lipoprotein profiles.

For the practical application of our model this implies the following strategy: First, vary the kinetic parameters of those processes known to be mostly affected by genetic variations (e.g. LDL uptake rate, LPL activity, rate of cholesterol transfer to HDL) and/or diet (synthesis rate of a composition of VLDL) in a physiologically reasonable range, calculate the associated LP profiles and store them in a lipoprotein profile data base. Second, compare the measured LP profile of a patient (the higher the resolution, the better) with all profiles in the data base and identify parameter constellations that would account for the patient's profile. Take this information as an adjunct to other independent findings to diagnose the molecular background of the patient's profile.

Finally, it has to be emphasized that the model can also be used to simulate the expected outcome of a proposed medical treatment following the diagnostic step described above.

### Conclusion

The model simulations successfully reproduce lipoprotein composition data of common density classes from healthy subjects and enable the revealing of the distribution of lipoproteins in high resolution. Abnormal distributions of lipoproteins can be predicted by modifying one of the underlying kinetic processes simulating lipid disorders. On the other hand, lipoprotein profiles of individual patients can be related to a selected set of kinetic parameters associated with abnormalities in the underlying processes of lipoprotein metabolism. In its present state, the model poses various questions to answer and offers a platform for many future applications aimed at understanding the reasons for inter-individual variability, identifying new sub-fractions of potential clinical relevance and a patient-oriented diagnosis of individual lipid abnormalities.

## Materials and Methods

### Stochastic Model

We consider a system of *N* lipoprotein complexes 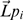, *i = (1, …, N)*, which are affected by *M* different kinetic processes *R_μ_*, *μ = (0, …, M)* in a unit volume *V*. Each lipoprotein complex 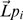 is unique with respect to its composition (*nA_i_*, *nB_i_*, *nF_i_*, *nC_i_*, *nT_i_)* where *nX_i_* is the number of molecules of component *X*, *X*∈{*A*,*B*,*F*,*C*,*T*}, in the lipoprotein *i*.

All lipoprotein complexes 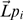 may be present with *n_i_* identical copies. The *n_i_* may be any non-negative integer number. As our model includes the exchange of the components A and F with plasma pools of free A and free F, respectively, and the exchange of the components C and T by the cholesteryl ester transfer protein (CETP) we also introduce the numbers *n_A_*, *n_F_*, *n_CETP(0)_*, *n_CETP(C)_* and *n_CETP(T)_* which denote the numbers of the respective component in the plasma pool. The state of the system is uniquely characterized by the vector 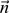 of all numbers *n_i_* and of all pool components.
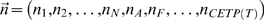
(1)The set of all thinkable vectors 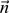 constitutes the *state space* of the system. Let 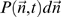 be the probability to observe the system in a small volume 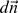 in the state space, i.e. the probability to find
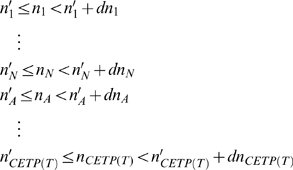
The function 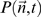 is the probability density of state 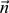. It contains all information about the evolution of the stochastic system over time. We denote with *N_from_* the set of states which may be transformed to state 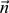 by a single reaction and with *N_to_* the set of all states which may be produced from 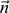 by a single reaction. Consider, for example the reaction *EffluxA_i_* representing the uptake of cholesteryl ester from an A-particle of type *i*. The event of this reaction would be to transform a particle of type *i* to type *i_-C_* which has one C less than *i*. Therefore, by action of the considered reaction the number *n_i_* is reduced by one. At the same time, the number 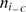 is increased by one (the total number of A-particles in the system is not affected by the considered reaction). Therefore, the set *N_to_* created by a reaction of type *EffluxA* (with arbitrary *i*) is the set of all states where one arbitrary A-particle is missing and in exchange, an A-particle with one less C is added. In the same manner the action of the other reactions has to be considered. The equation governing the evolution of the probability density 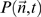
—the master equation—can be written
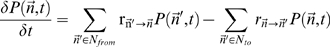
(2)Here, 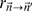 denotes the rate of the reaction transforming the state 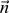 to 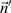. The explicit expression for this would be very complicated as we have to consider all possible results of the action of 20 different reaction types and will, therefore, be omitted here.

The Master equation (Equation 2) cannot be analytically solved. Therefore, we determined approximative numerical solutions by using Gillespie's stochastic simulation algorithm [51].

#### Gillespie's algorithm

The time evolution of the system is described as a sequence of events taking place at discrete time points. In each event, only one of the elementary processes is carried out instantaneously thereby changing the state of the system. The probability for reaction *μ* to occur next is proportional to its rate *a_μ_* which is considered constant between the events. The total probability rate *a*_0_ = Σ*a_μ_* is a measure for the total activity in the system. Two random numbers uniformly distributed over the unit interval are generated to determine both the waiting time *τ* until the next reaction occurs and the reaction *μ* which occurs after the previously determined waiting time. Reaction *μ* is characterized both by its type (e.g. EffluxA) and by the individual lipoprotein being its substrate. Execution of the reaction changes the state of the system (either by changing the number of lipoproteins or by altering the composition of one of them). Thus, recalculation of the reaction probabilities for the new state is needed, however, only for those *a_μ_* which were actually affected by the system change. The simulation time is advanced to *t* = *t*+*τ*. The process is repeated until the steady state of the system is reached or a different termination criterion is met. In our calculations this required approximately five millions of such consecutive single events. During the execution of the algorithm the lifetime of each lipoprotein in the system is monitored. This allows to calculate the average number of an individual lipoprotein complex in the steady state.

#### Deterministic model

If the probability density function 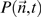 is known expectation values *c_i_(t)* for the concentration of lipoprotein complexes can be calculated according to
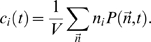
(3)The summation goes over all possible states of the system, i.e. over all legal combinations of *n_i_*.

Carrying out the calculation of the expectation values using the Master equation (Equation 2) one obtains a system of first-order differential equations for the time evolution of the concentration vector 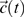:
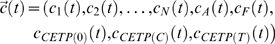
(4)The differential equation for the time-evolution of the concentration of the i-th lipoprotein complex has the general form (similar to the form of the master equation)
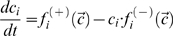
(5)where 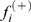 and 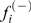 comprise all processes that increase or decrease the concentration *c_i_*, respectively. The stationary solution of this system obeys the fix-point equation Equation 6:
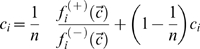
(6)which was solved iteratively. λ≥1 is an integer factor that helps to stabilize convergence, i.e. to overcome oscillations that may occur during iteration procedure.

### Density Profile Calculation

The density *d* of a lipoprotein complex is calculated as the sum of the component's molecular weights *w_i_* divided by the sum of the component's molecular volumes *v_i_*

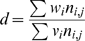
(7)where *i* specifies the components (A, B, F, C, T, P) and *n_i,j_* is the number of molecules of component *i* in the lipoprotein complex *j*. The number of phospholipid molecules is estimated to fill the calculated free volume within the lipoprotein surface (see Dataset S1). Values of the molecular weights and volumes were taken from literature and are listed in Table 4. From its amino acid composition, apoB-100 is estimated to have a molecular mass of 513 kDa. The somewhat higher apparent molecular mass (approximately 550 kDa) of the native protein is the result of glycosylation. For the lipid components CE, PL and TG we used average values because the molecular weight and volume may vary depending on the chain length and type (saturated, mono- or polyunsaturated) of the esterified fatty acids.

**Table 4:** Data for density calculation.

Component	Molecular Weight, g/mol	Molecular Volume, ml/mol	Reference
A	28,500	21,087	[59]
B	546,340	404,292	[60]
F	15,000	11,100	
C (FC+CE)	583	605	
T	859	947	[60]
P	786	773	[60]

The molecular weight of component F is averaged by taking individual molecular weights (see also [53]) of apoC isoforms, predominantly apoC-II (8.8 kDa) and C-III (8.9 kDa), and apoE (34 kDa) in a specific set ratio. Similarly, a 1:2 ratio for cholesterol:cholesteryl ester (with molecular weights of 386 and 648 Da, respectively) is used for the average molecular weight of component C. Molecular volumes were calculated using appropriate component's specific volumes [54].

### Experiments

#### Subjects

All laboratory assessments were performed at the Department of Clinical Chemistry, University of Freiburg, Germany. Lipid profiles of eleven randomly selected normolipidemic subjects were measured under fasting conditions. Normolipidemic concentration ranges of total plasma lipoprotein components are given in as follows: 120–240 mg/dl total cholesterol, 25–200 mg/dl triglycerides, 40–80 mg/dl free cholesterol, 80–160 mg/dl cholesteryl ester, 100–300 mg/dl phospholipids and 90–200 mg/dl for apolipoprotein A-I, 40–70 mg/dl A-II, 30–150 mg/dl B-100, 1–10 mg/dl C-II, 5–15 mg/dl C-III and 4–12 mg/dl E.

#### Lipoprotein separation

Lipoproteins were isolated from plasma by sequential preparative ultracentrifugation according to Baumstark [32].

Recoveries of cholesterol after centrifugation of all lipoproteins were >95%. The interassay coefficient of variance of the determination of apoB in each of the six LDL subfractions was ≤5% [8].

#### Lipoprotein chemistry

Cholesterol (C), triglyceride (T) and phospholipid (P) concentrations were determined enzymatically with the CHOD-PAP, GPO-PAP and PLD-PAP methods (Roche Diagnostics, Mannheim, Germany), respectively. Concentrations of apolipoproteins were determined by turbidimetry on a Wako 30 R analyzer (Wako Chemicals, Japan) using polyclonal antisera (Rolf Greiner Biochemica, Germany) specific for the respective antigens. For experimental details the reader is referred to [8].

### Parameter Estimation

Predicted and experimental lipoprotein profiles were compared by measuring the distance
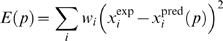
(8)where *p* is the vector of the model parameters. 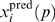 and 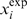 correspond to the simulated and measured concentrations of lipoprotein constituents in the i-th density class (see Table 2), respectively. *w_i_* is a weight that all the data points contribute equally to the distance. Model parameters are adjusted by minimizing the distance function (Equation 8). To avoid trapping of the minimization procedure in local minima we used Simulated Annealing (SA) as described in [52] to find the global optimum.

### Model Equations

For most reactions considered in the model, the exact kinetic mechanism including all regulatory effects is not known. Therefore, we used simple rate equations based on mass action kinetics. They are summarized in Dataset S4. There is a simple relationship between the values of the rate constants to be used in the stochastic and the deterministic model. The numerical values of rate constants of first-order reactions are identical in both types of models. In the case of second order reactions, the stochastic rate constant *c_μ_* derives from the deterministic rate constant *k_μ_* by
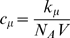
(9)where *N_A_* is the Avogadro constant and *V* denotes the small sample volume used in the stochastic simulation.

## Supporting Information

Dataset S1 Calculating the Number of Phospholipids
(0.03 MB PDF)

Dataset S2 Description of the Kinetic Processes Defined in the Model
(0.03 MB PDF)

Dataset S3 Explanations to the Model Parameter Values
(0.06 MB PDF)

Dataset S4 Model Equations
(0.03 MB PDF)
