# Supplementary material for: Computational Lipidology: Predicting Lipoprotein Density Profiles in Human Blood Plasma
Source: PLoS Comput Biol. 2008 May 23;4(5):e1000079. doi: 10.1371/journal.pcbi.1000079 (PMC2361219; doi:10.1371/journal.pcbi.1000079)
Supplement: Dataset S1 — Calculating the Number of Phospholipids (0.03 MB PDF) [file pcbi.1000079.s001.pdf]

Supplement S1. Calculating the Number of Phospholipids

The core volume V_c of a lipoprotein particle results from the number of neutral lipids n_i ($i = C, T$) and their molecular volume mv_i (in ml/mol) divided by the Avogadro constant L ($6.022 \cdot 10^{23}$ mol^{-1}) and is given as nm^3 per particle.

$$V_c = \sum_{i=\{C,T\}} n_i mv_i \cdot \frac{10^{21}}{L} \quad (1)$$

From the core volume the core radius r_c (in nm) can be calculated as follows. The surface radius r_s is assumed to have a fixed volume of $2nm$ [1]

$$r_c = \sqrt[3]{\frac{3}{4} \cdot \frac{V_c}{\pi}} \quad (2)$$

The surface volume V_s (in ml/mol) is then determined as:

$$V_s = 4 \cdot \pi \cdot r_c^2 \cdot r_s \cdot \frac{L}{10^{21}} \quad (3)$$

The surface of a lipoprotein is predominantly occupied by apolipoproteins and phospholipids. Thus, the surface volume occupied by phospholipids V_P is estimated as the difference of the total surface volume V_s and the surface volume already filled with apolipoproteins V_{AP}.

$$V_{AP} = \sum_{i=\{A,B,E\}} n_i mv_i \quad (4)$$

$$V_P = V_s - V_{AP} \quad (5)$$

Subsequently, the number of phospholipid molecules n_P is calculated.

$$n_P = \frac{V_P}{mv_P} \quad (6)$$

In the end, the density is recalculated by adding the weight and the volume for the phospholipid content.

$$d = \frac{\sum n_i mw_i + n_p mw_p}{\sum n_i mv_i + n_p mv_p} \quad (7)$$

References

1. Miller AL, Smith LC (1973) Activation of lipoprotein lipase by apolipoprotein glutamic acid. Formation of a stable surface film. J Biol Chem 248:3359–3362.
