# Supplementary material for: Computational Lipidology: Predicting Lipoprotein Density Profiles in Human Blood Plasma
Source: PLoS Comput Biol. 2008 May 23;4(5):e1000079. doi: 10.1371/journal.pcbi.1000079 (PMC2361219; doi:10.1371/journal.pcbi.1000079)
Supplement: Dataset S2 — Description of the Kinetic Processes Defined in the Model (0.03 MB PDF) [file pcbi.1000079.s002.pdf]

Supplement S2. Description of the Kinetic Processes Defined in the Model

Lp Species	Process	Description
A-particle	*Death and Birth*	
	CreateA	Synthesis and secretion of lipid-poor apoA-I molecules by the liver
	DestroyA	Receptor-mediated particle uptake, e.g. by the HDL receptor (HDLR)
	Lipoprotein-Tissue Exchange	
	Influx	Peripheral cholesterol is transferred to plasma HDL particles mediated by e.g. ABCA1
	EffluxA	Selective uptake of cholesteryl ester from HDL facilitated by e.g. SRB1
	Inter-Lipoprotein Exchange	
	ExchangeC_A	Cholesteryl ester is transferred from HDL to the non-lipid bound exchange protein ($CETP(0)$)
	ExchangeT_A	$CETP(T)$ releases its triglyceride content to HDL
	TransferA	Remodeling of apoA containing HDL particles by loosing and
	UptakeA	receiving a single molecule of apoA-I from the plasma pool
	TransferF_A	Delivery of apolipoproteins from HDL to newly synthesized VLDL via a free plasma pool
	UptakeF_A	Uptake of apolipoproteins by HDL particles
	Enzymatic Conversion	
	HydrolyzeA	Hydrolysis of triglycerides (release of free fatty acids) by the action of Hepatic Lipase (HL)
B-particle	*Death and Birth*	
	CreateB	Synthesis and secretion of VLDL by the liver
	DestroyB	Receptor-mediated particle uptake, e.g. by the LDL receptor (LDLR)
	Lipoprotein-Tissue Exchange	
	EffluxB	Selective uptake of cholesteryl ester from, e.g. LDL, facilitated by e.g. SRB1
	Inter-Lipoprotein Exchange	
	ExchangeC_B	$CETP(C)$ releases its cholesteryl ester to apoB-containing lipoproteins
	ExchangeT_{B1}	Triglyceride from apoB-containing lipoproteins is transferred to the non-lipid bound exchange protein ($CETP(0)$)
	ExchangeT_{B2}	$CETP(T)$ releases its triglyceride content to apoB-containing lipoproteins
	TransferF_B	Release of apolipoproteins during the delipidation cascade of apoB particles
	UptakeF_B	Uptake of further apolipoprotein molecules, e.g. of apoE by newly synthesized VLDL
	Enzymatic Conversion	
	HydrolyzeB	Hydrolysis of triglycerides (release of free fatty acids) by the action of Lipoprotein Lipase (LPL)

Abbreviations: CETP (Cholesteryl ester transfer protein), LPL (lipoprotein lipase), HL (hepatic lipase), ABCA1 (ATP-binding cassette transporter class A type 1), SRB1 (Scavenger receptor B1)
