# Supplementary material for: Computational Lipidology: Predicting Lipoprotein Density Profiles in Human Blood Plasma
Source: PLoS Comput Biol. 2008 May 23;4(5):e1000079. doi: 10.1371/journal.pcbi.1000079 (PMC2361219; doi:10.1371/journal.pcbi.1000079)
Supplement: Dataset S3 — Explanations to the Model Parameter Values (0.06 MB PDF) [file pcbi.1000079.s003.pdf]

Supplement S3. Explanations to the Model Parameter Values

Basically, in all processes (except birth and death of lipoprotein complexes) a lipoprotein component either newly enters or is removed from a lipoprotein complex. The rate depends on the amount of a kind of reservoir/transporter available in plasma and/or on the amount of the lipoprotein component in the appropriate lipoprotein complex, respectively. The underlying reaction mechanism can either be monomolecular (e.g. *EffluxA*) or bimolecular (e.g. $ExchangeC_A$) which is important to know while comparing the stochastic rate constant with parameters obtained from, e.g. tracer kinetic studies. In case of a monomolecular reaction both constants are equal. Since bimolecular reactions depend on the collision probability of both reaction partners the rate constant even depends on the volume in that the reaction takes place (Eq. 1).

$$c_\mu = \frac{k_\mu}{N_A \cdot V} \quad (1)$$

where c_μ and k_μ are the stochastic and kinetic rate constants of reaction μ, respectively. N_A is the Avogadro constant and V denotes the small sample volume used in the stochastic simulation. Most of the transfer and exchange processes in plasma are bimolecular.

We obtained a set of parameters that entail best agreement of the computed lipoprotein distribution with the experimental data. The estimated parameters agree in the order of magnitude compared to the kinetic data we found in the literature with some exceptions (see parameter table). For example, the estimated value for the selective uptake of HDL cholesteryl ester (CE), in our model called *EffluxA*, is a magnitude less than what is proposed in published data (0.01 vs. 0.31 day^{-1}, respectively) [1]. This might be caused by summarizing free cholesterol and cholesteryl ester in one component. The preferred physiological substrate of that efflux reaction is cholesteryl ester and the rate depends proportional on its concentration. Taking the sum of total cholesterol instead increases the substrate concentration available. To transport equal amounts of substrate (e.g. per day) out of the plasma a lower fractional catabolic rate would compensate a higher substrate concentration.

The comparison of calculated and measured rate constants for elevated processes such as the exchange of apolipoproteins, cholesteryl ester and triglycerides is difficult. In the model, the rate constants concern to elementary processes while kinetic measurements settle on compartment analyses. However, an attempt to relate those kinetic data to the estimated model parameters is proposed in the following for some examples. Each of them is marked in the parameter table with an appropriate index.

a) *Transfer of cholesteryl ester from HDL to apoB-100 carrying lipoproteins, e.g. VLDL, by the CETP.* This process is comparable to the bimolecular reaction we modeled in $ExchangeC_A$ which follows the rate law

$$v_{exchangeC_A} = c_{exchangeC_A} \cdot C \cdot CETP(0) \quad (2)$$

Jarnagin et al. characterized the specificity of a cholesteryl ester transfer protein from human plasma with a molecular weight of 74,000 Dalton [2]. The total transfer activity $v_{exchangeC_A}$ was assayed with 110.52 mg/dl $\cdot$ day^{-1} as the rate of loss of H^3-labeled cholesteryl ester in HDL (50 μg/ml in 0.5 ml incubation volume). The CETP mass is given with 0.049 mg/dl (= $6.6 \cdot 10^{-6}$ mmol/l). The kinetic rate constant $k_{exchangeC_A}$ equals according Eq. 2 the total transfer activity divided by the concentration of cholesteryl ester in HDL and by that CETP mass being in its non-lipid bound form $CETP(0)$ (in our calculations approximately 25.3 % of total CETP mass). Most of the CETP is loaded with C while the T-loaded form of CETP is very rarely with approximately 73.3 % and 1.4 %, respectively.

$$\begin{aligned} k_{exchangeCA} &= \frac{v_{exchangeCA}}{[CE] \cdot [CETP(0)]} \\ &= \frac{110.52(\text{mg/dl}) \cdot \text{day}^{-1}}{10\text{mg/dl} \cdot 1.67 \cdot 10^{-6}\text{mmol/l}} \\ &= 6.63 \cdot 10^6 \,\text{l/(mmol} \cdot \text{day)} \end{aligned}$$

According to Eq. 1 the kinetic rate constant is scaled to the volume (factor 60220 l/mmol) in that the simulation takes place and agrees in the order of magnitude to the calculated stochastic rate constant $c_{exchangeC_A}$ in our model (110.6 vs. 397.1 day^{-1}). However, the reference HDL-CE concentration is approximately one third of that in our simulation and the CETP mass is much less even. Thus, comparing the fluxes (total transfer activities) instead being 110.52 vs. 72.13 mg/dl $\cdot$ day^{-1} might be more useful.

b) *Transfer of triglycerides (TG) from VLDL to, e.g. HDL by the CETP*. Similarly, Jarnagin et al. provide the rate value of this process relative to the CE transfer (0.11 nmol TG relative to 1 nmol CE per ml per h). We modeled this bimolecular process in $ExchangeT_{B1}$ which follows the rate law

$$v_{exchangeT_{B1}} = c_{exchangeT_{B1}} \cdot T \cdot CETP(0) \quad (3)$$

The total transfer activity $v_{exchangeT_{B1}}$ is 15.87 mg/dl $\cdot$ d^{-1}, accordingly. From the given CE (100 μg in 0.5 ml incubation volume) to TG ratio for VLDL (0.15) follows the VLDL-TG concentration of 133.3 mg/dl. By taking the CETP(0) mass given above the (volume scaled) kinetic rate constant is more than two magnitudes less than the estimated model parameter (1.2

vs. 887.75 day^{-1}). However, in this case the reference VLDL-TG concentration is approximately double of that in our simulation and even the CETP mass is less. Thus again, comparing the fluxes (total transfer activity) instead being 15.87 vs. 297.45 mg/dl · day^{-1} might be more useful.

In general, one major reason for discrepancies in these processes might be due to the fact that we modeled the exchange of CE and TG uncoupled. That means that e.g. triglycerides (component T) of B-particles can be transferred as long as triglycerides and an appropriate acceptor (non-lipid bound CETP) are available independent on the amount of CE (component C) in A-particles.

The parameters for the transfer and uptake processes of component F are only slightly interpretable with the current state of our model because we do not specify a particular apolipoprotein. However, Batal et al. investigated the plasma kinetics of VLDL and HDL apoC-III and apoE, both are potential candidates for component F [3].

c) *Transfer of apolipoprotein F (apoC+apoE) from HDL.* We modeled this process in $TransferF_A$ which follows the rate law

$$v_{transferF_A} = c_{transferF_A} \cdot F \cdot poolF(0) \tag{4}$$

Batal et al. have proposed fractional catabolic rates (FCR) of both apolipoprotein (apo) CIII and E in HDL with 0.285 and 1.1 day $^{-1}$, respectively. The sum of concentration of apoC-III (5.34 mg/dl) and apoE (2.99 mg/dl) is 8.33 mg/dl. Subsequently, the total transfer rate of component F is the sum of the transfer rates of apoC-III (1.52 mg/dl per day) and apoE (3.29 mg/dl per day) = 4.81 mg/dl per day. This value includes two elementary processes by which the apolipoproteins can disappear: i) by the receptor-mediated uptake of HDL and ii) by the selective transfer out of HDL. The uptake rate of F can be calculated from the FCR (0.2 day^{-1}) and concentration of HDL apoA-I (118 mg/dl). By taking the proportion of F relative to apoA-I in a HDL particle we get a HDL apoF uptake rate of 0.22 mg/dl per day. Accordingly, the transfer rate out of HDL (difference between the total and the uptake rate) and the appropriate FCR are 4.59 mg/dl · day^{-1} and 0.551 day^{-1}, respectively. In the model, the process is formulated in that the monomolecular transfer reaction also depends on the capacity of the plasma to accept a further free apolipoprotein of type F ($poolF(0)$). The literature reference value, however, does not consider this factor but can taken from our calculations (1.2e-3 mmol/l). Thus, the kinetic constant is divided by that factor yielding an experimental value that agree in one order of magnitude less with the simulated parameter value (459.2 vs. 56.9 l/mmol per day). The volume scaled (factor of 60220 l/mmol) values are 7.6e-3 and 9.4e-4 day^{-1}, accordingly. Since this process is monomolecular we scale both constants equally.

d) *Uptake of apolipoprotein F (apoC+apoE) by HDL.* Apolipoproteins can also newly enter a lipoprotein complex, e.g. an A-particle. In our model, this bimolecular process is called $UptakeF_A$ and follows the rate law

$$v_{uptakeF_A} = c_{uptakeF_A} \cdot poolF \tag{5}$$

Batal et al. [3] provide a transfer rate (TR) for HDL apoC-III and apoF of 0.8 mg/kg·day^{-1} and 1.56 mg/kg·day^{-1}, respectively. Thus, for both apolipoproteins the total transfer activity being 5.24 mg/dl·day^{-1} (0.45 dl/kg body weight). This value again comprises two processes by which apolipoproteins can enter a lipoprotein complex: i) as components of newly synthesized A-particles and ii) by the selective uptake from a free plasma pool. Since in our model nascent A-particles are free of apoC and apoE the total transfer activity equals the uptake activity from a free plasma pool. Thus, the kinetic rate constant yields the value of 4.77 day^{-1} by dividing the uptake activity by the concentration of the free plasma pool (approximately 1.1 mg/dl). However, this experimental rate constant does not taking int account the amount of lipoprotein complexes being available as acceptor molecules in the plasma. In the simulation approximately 0.02 mmol/l of A-particles are present by which the kinetic rate constant is divided. According to Eq. 1 the kinetic rate constant is scaled to the volume (factor 60220 l/mmol) in that the simulation takes place and agrees in the order of magnitude with the estimated stochastic rate constant (3.9e-3 vs. 1.9e-3 day^{-1}).

e) *Transfer of apolipoprotein F (apoC+apoE) from VLDL.* We modeled this process in $TransferF_B$ which follows the rate law

$$v_{transferF_B} = c_{transferF_B} \cdot F \cdot poolF(0) \tag{6}$$

FCR of apoC-III and VLDL apoE in VLDL proposed by Batal et al. [3] are 0.85 and 4.76 day^{-1}, respectively. Concentrations are 3.59 mg/dl VLDL apoC-III and 0.72 mg/dl VLDL apoE - in sum 4.31 mg/dl. The total transfer activity for both apolipoproteins is 6.48 mg/dl per day. Assuming that the selective transfer of the apolipoproteins takes a minor part the rate was set to 0.5 mg/dl per day. The kinetic rate constant is then divided by the factor $poolF(0)$ (see c)) and agrees well to the model parameter value (96.67 vs. 120.54 l/mmol per day). The volume scaled (factor of 60220 l/mmol) values are 1.6e-3 and 2.0e-3 day^{-1}, accordingly. Since

this process is monomolecular we scale both constants equally.

f) *Uptake of apolipoprotein F (apoC+apoE) by VLDL.* In our model, this bimolecular process is called $UptakeF_B$ and follows the rate law

$$v_{uptakeF_B} = c_{uptakeF_B} \cdot poolF \quad (7)$$

Batal et al. [3] provide a transfer rate (TR) for VLDL apoC-III and apoF of 1.35 mg/kg $\cdot day^{-1}$ and 1.59 mg/kg $\cdot day^{-1}$, respectively, yielding a total transfer activity of 6.53 mg/dl $\cdot day^{-1}$ (0.45 dl/kg body weight). The rate of selective uptake of component F from the plasma pool (difference of total transfer activity and fractional synthesis) is assumed to be approximately half of the total synthesis rate (=3.25 mg/dl per day). According to d) the kinetic rate constant is obtained by: i) dividing the selective uptake activity by the concentration of the free 'apoF' in plasma ($poolF$ approximately 1.1 mg/dl), ii) dividing the amount of lipoprotein complexes being available as acceptor molecules in the plasma (in the simulation 8.04e-4 mmol/l) and iii) scaling to the volume (factor 60220 l/mmol) in that the simulation takes place. Finally, the experimental value agrees in one order of magnitude less to the simulated parameter value (0.061 vs. 3.5e-3 day^{-1})

g) *Transfer of apolipoprotein A from HDL.* We modeled this process in $TransferA$ which follows the rate law

$$v_{transferA} = c_{transferA} \cdot A \cdot poolA(0) \quad (8)$$

Cohn et al. [4] provide FCR and total transfer activity of HDL apoA-I of 0.196 day^{-1} and 24.44 mg/dl per day. The concentration of HDL apoA-I is given with 118.4 mg /dl. According to c) the transfer activity includes two elementary processes by which apoA-I can disappear: i) by the receptor-mediated uptake of HDL and ii) by the selective transfer out of HDL. The receptor-mediated uptake rate is 23.13 mg/dl per day (FCR times concentration) yielding the selective transfer rate of 1.31 mg/dl per day (difference of total transfer rate and receptor-mediated uptake rate). According to Eq. 8, the kinetic rate constant is obtained by: i) dividing the concentration of HDL apoA-I, ii) dividing by the factor $poolA(0)$ being the capacity of the plasma to accept a further free apolipoprotein A-I (in our simulation 0.002 mmol/l). Finally, the experimental value agrees in the order of magnitude to the model parameter value (5.53 vs. 12.62 l/mmol per day). The volume scaled (factor of 60220 l/mmol) values are 9.2e-5 and 2.0e-4 day^{-1}, accordingly. Since this process is monomolecular we scale both constants equally.

h) *Uptake of apolipoprotein A by HDL.* In our model, this bimolecular process is called $UptakeA$ and follows the rate law

$$v_{uptakeA} = c_{uptakeA} \cdot poolA \quad (9)$$

HDL apoA-I total transfer activity of 24.44 mg/dl per day is taken from [4]. This value includes i) apoA-I as component of newly synthesized A-particles and ii) the selective uptake of apoA-I from a free plasma pool. In the model, newly synthesized particles contain two apoA-I molecules. Together with the synthesis rate of 4e-3 mmol/l per day and the molecular weight of 28500 g/mol we get a selective synthesis rate of 22.8 mg/dl per day. Thus, the selective uptake rate from the plasma is 1.64 mg/dl per day (difference of total transfer activity and selective synthesis rate). The kinetic rate constant is obtained by: i) dividing the selective uptake rate by the concentration of the free 'apoA' in plasma (in the model $poolA$ approximately 0.01 mg/dl), ii) dividing the amount of lipoprotein complexes being available as acceptor molecules in the plasma (in the simulation 0.02 mmol/l) and iii) scaling to the volume (factor 60220 l/mmol) in that the simulation takes place. Finally, the experimental value agrees in one order of magnitude less to the simulated parameter value (0.136 vs. 0.02 day^{-1}).

For processes such as $EffluxB$, $ExchangeT_A$, $ExchangeC_B$ and $ExchangeT_{B2}$ no suitable reference values could be found.

References

1. Ouguerram K, Krempf M, Maugeais C, Maugére P, Darmaun D, et al. (2002) A new labeling approach using stable isotopes to study in vivo plasma cholesterol metabolism in humans. Metabolism 51:5–11.

2. Jarnagin AS, Kohr W, Fielding C (1987) Isolation and specificity of a Mr 74,000 cholesteryl ester transfer protein from human plasma. Proc Natl Acad Sci 84:1854–1857.

3. Batal R, Tremblay M, Barrett P, Jacques H, Fredenrich A, et al. (2000) Plasma kinetics of apoC-III and apoE in normolipidemic and hypertriglyceridemic subjects. J Lipid Res 41:706–718.

4. Cohn JS, Batal R, Tremblay M, Jacques H, Veilleux L, et al. (2003) Plasma turnover of HDL apoC-I, apoC-III, and apoE in humans: in vivo evidence for a link between HDL apoC-III and apoA-I metabolism. J Lipid Res 44:1976–1983.
