# Supplementary material for: Computational Lipidology: Predicting Lipoprotein Density Profiles in Human Blood Plasma
Source: PLoS Comput Biol. 2008 May 23;4(5):e1000079. doi: 10.1371/journal.pcbi.1000079 (PMC2361219; doi:10.1371/journal.pcbi.1000079)
Supplement: Dataset S4 — Model Equations (0.03 MB PDF) [file pcbi.1000079.s004.pdf]

Supplement S4. Model Equations

A-particle		
$v_{createA}$	=	$v_{createA} = const$
$v_{destroyA}$	=	$c_{destroyA} \cdot X_i$
v_{influx}	=	$v_{influx} = const$
$v_{effluxA}$	=	$c_{effluxA} \cdot nC_i$
$v_{exchangeC_A}$	=	$c_{exchangeC_A} \cdot nC_i \cdot CETP(0)$
$v_{exchangeT_A}$	=	$c_{exchangeT_A} \cdot CETP(T)$
$v_{transferA}$	=	$c_{transferA} \cdot nA_i \cdot (A_{max} - A)$
$v_{uptakeA}$	=	$c_{uptakeA} \cdot A$
$v_{transferF_A}$	=	$c_{transferF_A} \cdot nF_i \cdot (F_{max} - F)$
$v_{uptakeF_A}$	=	$c_{uptakeF_A} \cdot F$
$v_{hydrolyzeA}$	=	$c_{hydrolyzeA} \cdot nT_i$

B-particle		
$v_{createB}$	=	$v_{createB} = const$
$v_{destroyB}$	=	$c_{destroyB} \cdot X_i$
$v_{effluxB}$	=	$c_{effluxB} \cdot nC_i$
$v_{exchangeC_B}$	=	$c_{exchangeC_B} \cdot CETP(C)$
$v_{exchangeT_{B1}}$	=	$c_{exchangeT_{B1}} \cdot nT_i \cdot CETP(0)$
$v_{exchangeT_{B2}}$	=	$c_{exchangeT_{B2}} \cdot CETP(T)$
$v_{transferF_B}$	=	$c_{transferF_B} \cdot nF_i \cdot (F_{max} - F)$
$v_{uptakeF_B}$	=	$c_{uptakeF_B} \cdot F$
$v_{hydrolyzeB}$	=	$c_{hydrolyzeB} \cdot nT_i$

v_μ and c_μ are rate and rate constant of reaction μ, respectively; nX_i denotes the amount of component X, X=(A,B,F,C,T) in a lipoprotein complex i; X_i is the number of lipoprotein i in the system; A_{max}, F_{max} and A, F are the maximal and actual number of free A and F in the plasma pool, respectively. $CETP(0)$ and $CETP(C)$, $CETP(T)$ denote the non-lipid bound and lipid bound (either with C or T) transport forms of the cholesteryl ester transfer protein (CETP), respectively.
